# Molecular vibrations reduce the maximum achievable photovoltage in organic solar cells

**DOI:** 10.1038/s41467-020-15215-x

**Published:** 2020-03-20

**Authors:** Michel Panhans, Sebastian Hutsch, Johannes Benduhn, Karl Sebastian Schellhammer, Vasileios C. Nikolis, Tim Vangerven, Koen Vandewal, Frank Ortmann

**Affiliations:** 1 Center for Advancing Electronics Dresden, Technische Universität Dresden, 01062 Dresden, Germany; 2 Dresden Integrated Center for Applied Physics and Photonic Materials (IAPP) and Institute for Applied Physics, Technische Universität Dresden, Nöthnitzer Str. 61, 01187 Dresden, Germany; 3 Institute for Materials Research (IMO-IMOMEC), Hasselt University, Wetenschapspark 1, 3590 Diepenbeek, Belgium

**Keywords:** Excited states, Solar cells, Condensed-matter physics

## Abstract

The low-energy edge of optical absorption spectra is critical for the performance of solar cells, but is not well understood in the case of organic solar cells (OSCs). We study the microscopic origin of exciton bands in molecular blends and investigate their role in OSCs. We simulate the temperature dependence of the excitonic density of states and low-energy absorption features, including low-frequency molecular vibrations and multi-exciton hybridisation. For model donor-acceptor blends featuring charge-transfer excitons, our simulations agree very well with temperature-dependent experimental absorption spectra. We unveil that the quantum effect of zero-point vibrations, mediated by electron-phonon interaction, causes a substantial exciton bandwidth and reduces the open-circuit voltage, which is predicted from electronic and vibronic molecular parameters. This effect is surprisingly strong at room temperature and can substantially limit the OSC’s efficiency. Strategies to reduce these vibration-induced voltage losses are discussed for a larger set of systems and different heterojunction geometries.

## Abstract (web summary)

A steep absorption edge is preferred for high performance solar cells, but is less common for organic solar cells (OSCs). Here Panhans et al. find that the absorption tails are dominated by zero point vibrations and are responsible for the lowering of the open-circuit voltage and the performance of OSCs.

## Introduction

Studies of UV–Vis–NIR absorption for conventional semiconductors usually focus on the absorption strength and the energy of spectral features. This is similar for organic semiconductors, which exhibit strong excitonic effects^1^ and for which the nature of the excitons (molecular excitons, charge-transfer (CT) excitons or hybrids) and their connection to performance limitations of optoelectronic devices are of great interest^2–7^. For instance, the physical mechanisms and microscopic interactions that influence the key electron-transfer events in OSCs are broadly debated^1,3,8–12^ and can be studied by absorption spectroscopy. Important characteristics of the spectra are the lineshape and the width of the absorption bands, whose low-energy tail determines the maximum achievable open-circuit voltage of the material system. Temperature-dependent exponential absorption tails, so-called Urbach tails^13^, have been frequently found in conventional semiconductors such as GaAs^14^—a high-performing photovoltaic material. More recently, in conjugated polymers, absorption tails with linewidths or Urbach energies^15–18^ in the range of *k*_B_*T* at room temperature have been reported. Investigating the temperature dependence of such bands would be of particular importance because the excitonic processes in these systems, including absorption, depend on both the strong electron–phonon coupling (EPC), induced by molecular vibrations^19^, and the ubiquitous disorder^15,16^ either of which are assumed to dominate the exciton bandwidth on the low-energy side. However, up to now, neither the lineshape nor its microscopic origin are well understood in polymer- or small-molecule-based blends.

To investigate the impact of microscopic molecular parameters on lineshape, linewidth and thermal disorder, as well as their influence on OSC device parameters, we study theoretically and experimentally model systems of electron donating (donor) and electron accepting (acceptor) molecules. Based on the simulation of excitonic properties (including molecular vibrations and exciton hybridisation) and sensitively measured external quantum efficiency (EQE) spectra for temperatures between 80 and 350 K, we find that absorption tails can be dominated by zero-point vibrations^20^ even at room temperature. This fundamental quantum effect is responsible for lowering the performance of OSCs, thus rendering vibrations and their EPC an important subject for future OSC research. When pure electronic optimisation strategies, such as reducing the electronic driving energy, are exhausted^21,22^, voltage losses can be reduced by lowering the molecular vibrational coupling to low-frequency vibrations or increasing the electronic/excitonic coupling between states. Both directions lead to steeper absorption tails and mitigate the impact of this quantum effect towards higher solar cell efficiencies.

## Results

### Theoretical approach

We want to model the low-energy optical absorption of OSC materials, which is strongly influenced by the electron–electron and the electron–phonon interaction that lead to excitonic states that are spatially more localized than Wannier excitons in traditional semiconductors. To include excitonic effects, we need to simulate the excitonic density of states (EDOS). The prediction of the EDOS takes into account the electronic and vibrational properties of the organic systems, which we describe with the Hamiltonian

1 $$H = H_{{\mathrm{el}}} + H_{{\mathrm{el}} - {\mathrm{el}}} + H_{{\mathrm{el}} - {\mathrm{ph}}} + H_{{\mathrm{ph}}},$$

where *H*_el_ describes the orbital energies and electronic couplings via transfer integrals in a molecular site basis. We study the low-energy excitations and can restrict ourselves to the states derived from the highest occupied molecular orbitals (HOMO) and lowest unoccupied molecular orbitals (LUMO) of donor and acceptor molecules. *H*_el−el_ is the Coulomb-type electron–hole interaction, *H*_ph_ describes the molecular vibrations and *H*_el−ph_ their EPC^23,24^ (see Supplementary Methods for details).

Analogous to the conventional electronic density of states that describes the energetic distribution of charged excitations (by adding electrons or holes in bulk organic films)^25–28^, we simulate the EDOS

2 $$D\left(\omega \right) = \mathop {\sum }\limits_{hl} \mathop {\sum }\limits_i \langle i|\left[{\rho a_la_h^\dagger \delta \left({\hbar \omega + E^i - H} \right)a_ha_l^\dagger } \right]\left| i \right.\rangle,$$

which describes charge-neutral excitations. Its temperature dependence, which will be discussed below, enters Eq. (2) through the statistical operator *ρ* and the sum runs over initial vibrational and electronic states |*i*〉 of energy *E*^*i*^. Note that this definition is analogous to an absorption coefficient apart from the matrix elements for optical transitions (see Supplementary Methods).

In order to study the sub-gap lineshape of the CT EDOS in organic blends, we need to describe the effect of low-frequency vibrations in microscopic detail^29^. Here, a convenient simplification is to describe the skeletal intra-molecular modes (high-frequency vibrations with typical mode energies *ħω*_hf_ > 125 meV) by their vibrational ground states because of the negligibly small population of these modes (*k*_B_*T* ≪ *ħω*_hf_) at room temperature and below. This allows avoiding unnecessary complexity arising from phonon replica in the present study since their EPCs are not large^19^. The low-frequency vibrations enter the thermal average for the EDOS (see Supplementary Methods)

3 $$D({\omega} ) = {\sum}_{{hl}\ {{el}}}\left\langle 0 \right| a_l a_{h}^{\dagger} \delta \left({\hbar} \omega - H^{\prime} - V\left( T \right) \right) a_{h} a_{l}^{\dagger} \left| 0 \right\rangle_{{el}} ,$$

which depends only on the reduced Hamiltonian $H^{\prime}=H-H^{\mathrm{lf}}_{\mathrm{ph}}-H^{\mathrm{lf}}_{\mathrm{el}-\mathrm{ph}}$ that still includes all electronic and excitonic coupling terms. In particular the hybridisation between molecular and CT excitons due to electron and hole transfer integrals can be described. The low-frequency vibrations manifest in a vibrational sub-manifold for the exciton states and can be described by the concept of thermal disorder *V*(*T*). Its strength is related to their molecular EPC parameters. We emphasise that this analytical result is exact in the limit of low vibration frequencies. It is central to an efficient treatment of these modes. This thermal disorder can be superimposed with conventional static disorder *V*_D_, which may add an inhomogeneous disorder contribution.

### Simulation of EDOS and its low-energy tails

**Fig. 1:**
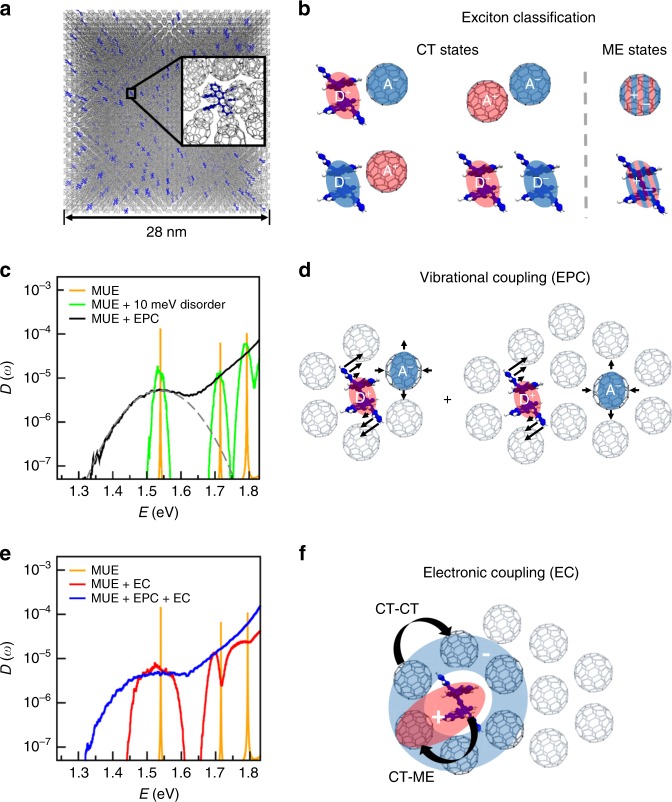
First model system, excitonic density of states and schematics of excitonic states. **a** Perspective view to a rubrene:C_60_ blend with a system size of around 40,000 molecules used for the EDOS simulations. Inset: zoom to a molecular cluster with a rubrene molecule as donor (ball and stick model) embedded in C_60_ fullerene host molecules (stick model). Simulations are performed for 6 mol% donor concentration in a C_60_ host. **b** Classification of excitons that are included in the simulations and contribute to the EDOS. “A” denotes the acceptor and “D” the donor compound, respectively. For simplicity, the charge separated states are included in the class of CT states (at large distance) and are not distinguished further in this scheme but also contribute to the EDOS. All the non-interacting electron–hole pairs considered here define the manifold of uncoupled excitons (MUE). **c**, **e** EDOS of different exciton models based on the rubrene:C_60_ system to demonstrate different broadening mechanisms (disorder, electronic/excitonic coupling (EC) and EPC; see Supplementary Methods). **d**, **f** Illustration of the impact of different interactions (EPC, EC) on the states in the MUE. **c** Orange lines: EC and EPC are set to zero (MUE model). The peaks appear at analytical CT energies thus validating the numerical approach. The heights reflect the state degeneracies. The assumed numerical width is sufficiently small and not relevant. Green line: inhomogeneous disorder (*σ*_D_ = 10 meV for illustration purpose) broadens the CT-peaks of the MUE. Black line: room-temperature EDOS (*T* = 300) including EPC, which induces a thermal linewidth of *σ*_loc_ = 69 meV. Dotted grey line: Gaussian fit to the EDOS with $\sigma^{\mathrm{num}}_{\mathrm{loc}}=71$ meV. **e** EDOS including EC between molecules assuming random C_60_ orientation and random distribution of transfer integrals (Gaussian distributed with *σ*_TI_ = 17 meV) and *V*_D_ = 0. Red line: EDOS with zero EPC (*V*(*T*) + *V*_D_ = 0) yields a CT half-bandwidth of 90 meV. Blue line: EDOS including all microscopic interactions (EC and EPC); Orange lines indicate analytical energies from **c**.

In the first part of our study, we focus on small-molecule donor–acceptor blends with a low donor concentration of 6 mol%, where the molecules are randomly distributed in the host^30^ for convenience of modelling. We demonstrate the emergence of CT tails for the first model system rubrene:C_60_. Its EDOS is calculated numerically from Eq. (3) with a Lanczos approach^31^ (see “Methods”) in Fig. 1. To the ease of the reader, we first discuss a purely electronic (i.e. vibration-less) model of the rubrene:C_60_ blend that has zero exciton bandwidth henceforth denoted manifold of uncoupled excitons (MUE) and subsequently study three bandwidth broadening mechanisms (details are given in Supplementary Methods). The EDOS of the MUE exhibits sharp peaks (Fig. 1c), which correspond to the discrete sub-manifold of uncoupled low-energy donor–acceptor CT excitons.

To study excitonic interactions, we investigate the broadening of CT exciton bands in bulk D-A systems. This broadening is the quantity of interest and needs to be distinguished from the residual broadness of zero-phonon lines in recent single-molecule spectroscopy experiments^32^ (see Supplementary Methods for a detailed discussion). The broadening of CT exciton bands can be induced by three mechanisms. Firstly, conventional static disorder may lead to an inhomogeneous broadening (for illustration purpose we choose *V*_D_ = 10 meV in the simulations plotted in Fig. 1c). Secondly, we study the vibration-induced mechanism by low-frequency vibrations by including their EPC in the simulations (henceforth denoted MUE + EPC). In this model, we obtain a strong broadening effect due to the thermal molecular motion (Fig. 1c). The excitonic states that dominate the EDOS at low energies and the impact of the EPC on these states are schematically illustrated in Fig. 1d, where the black arrows indicate the vibrational modes of the rubrene and the C_60_ molecule. The third mechanism is the electronic and/or excitonic coupling (EC) between states (henceforth denoted MUE + EC). This is simulated by describing the full hybridisation between CT states (CT–CT coupling) and between CT and molecular excitons (CT–ME coupling)—a substantial extension to three-state models achieved in recent literature. Here, the exciton band is a consequence of the high connectivity of the C_60_ molecules surrounding the donor, which is illustrated in Fig. 1f. Previous experiments have suggested that electrons can rapidly delocalise in a related set of CT states hosted by C_60_ clusters^2,9^ and also prior theoretical studies have emphasised the role of molecular aggregation^33^. Intriguingly, the tail at the low-energy side of this simulated CT band resembles an exponential rather than a Gaussian lineshape that occurred in MUE + EPC.

We turn to the full description of the blends and study—in absence of disorder *V*_D_—the interrelation of electronic coupling and thermal vibrational disorder from EPC (blue line in Fig. 1e). In contrast to the exponential low-energy CT tail of MUE + EC, it exhibits a Gaussian tail. Quite surprisingly, we find that the extracted standard deviation of this tail is not increased upon combining the two broadening mechanisms but slightly decreased (*σ*_tot_ = 69.7 meV) compared with MUE + EPC despite the presence of random EC. This leads us to the conclusion that the vibrations and their thermal disorder dominate the absorption linewidth at the low-energy tail for which we predict a Gaussian shape.

### Sensitive EQE measurements and temperature-dependent tails

In order to study these effects experimentally and to validate the simulations, we perform ultra-sensitive EQE spectroscopy on the rubrene:C_60_ system and other donor-C_60_ systems (see Supplementary Fig. 2b for an illustration of all compounds and “Methods” for experimental details). All donor-C_60_ systems are prepared at 6 mol% donor diluted in the C_60_ host. The comparison of the low-energy region (sub-gap absorption) of the blended systems to the absorption of pristine C_60_ films (whose excitonic gap is above 1.8 eV) clearly indicates the intermolecular CT character of the absorption bands in the blends, which are centred at about 1.3–1.5 eV.

**Fig. 2:**
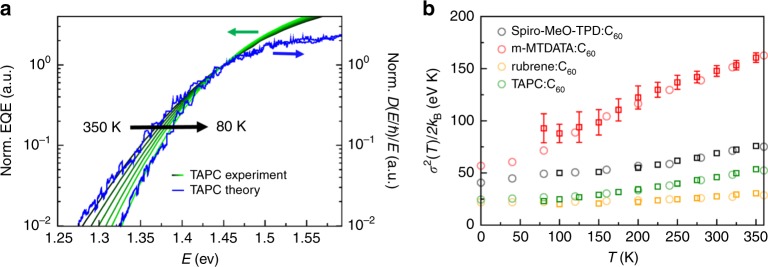
Temperature-dependent CT bands and linewidths. **a** Simulated EQE for TAPC:C_60_ (6 mol% donor blend) at the temperatures 80 and 350 K (blue lines) in comparison with experimental EQE spectra for different temperatures from 80 to 350 K (green with different shades). All curves are normalised at *E* = 1.45 eV. **b** Simulated linewidth for TAPC:C_60_ and three additional donor:C_60_ combinations extracted from numerical spectra (circles) and linewidths of measured EQE spectra (squares), extracted from the data in Supplementary Figs. 3–10. Error bars indicate estimated uncertainties in the experimental fit procedure. For clarity, no error bars are plotted if the fit uncertainty (in theory and experiment) is smaller or equal to the symbol size.

To identify the dominant broadening effect, we study theoretically and experimentally the temperature dependence of the EQE spectrum for TAPC:C_60_ for temperatures between 80 and 350 K in Fig. 2a. In the simulations, the low-energy band tails exhibit a Gaussian lineshape whose linewidth is significantly reduced when reducing the temperature down to 80 K—a direct consequence of freezing the vibrations. The corresponding temperature-dependent EQE measurement at the same donor concentration shows also a strong narrowing of the absorption tails upon cooling, which would not occur for disorder-induced bands (i.e. inhomogeneous broadening). The absence of inhomogeneous disorder in the simulations and the close agreement for the temperature-dependent spectral tails leads us to the conclusion that indeed the vibrations with their couplings are the dominant source of the CT exciton bandwidth in the studied system and the influence of disorder is rather small. We note that small disorder for charge-neutral excitations is not in conflict with possible larger disorder that could occur for charges (reflected in the conventional DOS) or for other blend compositions or materials.

The close agreement between experiment and theory for the large temperature range is confirmed in Fig. 2b for a larger set of systems comprising donor molecules: rubrene, m-MTDATA, Spiro-MeO-TPD and TAPC (see Supplementary Methods for molecular structures), thus demonstrating the generality of these findings and the ability of the approach to describe efficiently the microscopic physical interactions in these systems. The donor molecules in this set are chosen to have different size and conformational (or vibrational) flexibility. Indeed, the donors with the largest EDOS tails (Spiro-MeO-TPD and m-MTDATA) are known for their flexible dynamics at room temperature as compared for instance with rubrene. This requires conformational sampling of the flexible donor:C_60_ pairs by simulating the thermal motion with molecular dynamics simulations of molecular clusters (see Supplementary Methods).

### Lower bound for thermal disorder

Despite the different magnitudes for the width of the EDOS tails of different materials, each donor:C_60_ system shows a reduced broadening *σ*(*T*) when cooling down to 80 K, which indicates reduced thermal disorder (Fig. 2b). Interestingly, in absence of inhomogeneous disorder in the simulations (*V*_D_ = 0), all systems exhibit a finite broadness at low temperature. This is also a universal feature for all studied donor molecules in our experimental work in which we were able to measure EQE spectra at liquid nitrogen temperatures. The low-temperature value of the tail broadness varies between different donors. This residual width at low *T* is caused by the slow vibrational modes, which lead to a zero-point vibration effect. That is, at zero temperature, quantum fluctuations of the low-frequency vibrations induce an effective broadening that is of fundamental quantum nature. This sets a lower bound of the thermal disorder, which causes a width of the band tail that remains even if all other sources of inhomogeneous disorder can be avoided. For all studied systems, thermal disorder describes the experiment accurately down to the lowest accessible temperatures. Substantial inhomogeneous disorder would increase the tail broadness beyond this value, which would be inconsistent with the measurements. Surprisingly, this quantum effect is not restricted to 0 K. In contrast, even at room temperature, one observes a broadness for rubrene and TAPC that is similar to the one at absolute zero, evidencing that zero-point fluctuations may contribute significantly to the absorption tails of organic devices even at ambient conditions. Indeed, at *T* = 300 K we determine their relative contribution to $\frac{{\sigma \left( {T \, = \, 0{\mathrm{K}}} \right)}}{{\sigma \left( {T \, = \, 300{\mathrm{K}}} \right)}} = 60{\hbox{-}}85\%,$, i.e. they dominate the tails for the systems shown in Fig. 2.

### Impact on the radiative limit of the open-circuit voltage

The above findings have direct consequences for the efficiency of OSCs since the zero-point vibrations impact the low-energy tail of the absorption that is connected to the open-circuit voltage (*V*_oc_)—a crucial quantity for their efficiency^34–36^. We simulate the influence of the absorption linewidth on *V*_oc_ in the radiative limit (denoted as *V*_r_), which is the thermodynamic upper limit of *V*_oc_ considering only radiative recombination^37^. A solar cell’s *V*_r_ at temperature *T* is described by the relation^38^

4 $$V_{\mathrm{r}}(T) = \frac{{k_{\mathrm{B}}T}}{q}\ln \left( {\frac{{{\int} {dE\phi _{{\mathrm{Sun}}}\left( E \right)\Sigma \left( {E} \right)} }}{{{\int} {dE\phi _{{\mathrm{BB}}}\left( {E,T} \right)\Sigma \left( {E} \right)} }} + 1} \right),$$

with the solar spectrum *ϕ*_Sun_(*E*) and the thermal black-body-radiation spectrum *ϕ*_BB_(*E,T*). Σ(*E*) is the photovoltaic quantum efficiency spectrum^37^.

**Fig. 3:**
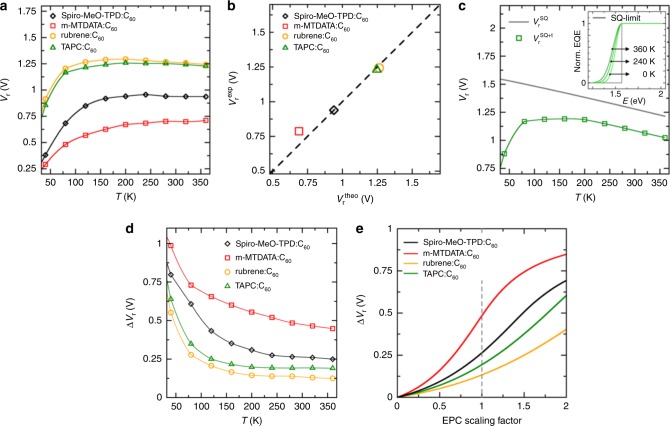
Radiative limit of the open-circuit voltage and vibration-induced radiative voltage losses. **a** *V*_r_ for different donor:C_60_ blends based on the simulated EDOS *D*(*ω*) and the AM1.5G solar spectrum (Eq. 4). Donor species are indicated. **b** Comparison of theoretical and experimental *V*_r_ at room temperature. Experimental room-temperature data are taken from ref. ^54^. **c** Comparison of $V_{\mathrm{r}}^{\mathrm{SQ}}$ of the Shockley–Queisser (SQ) model (grey) to $V_{\mathrm{r}}^{\mathrm{SQ}+\mathrm{t}}$ of a broadened SQ model based on the TAPC:C_60_ tail (green). Inset: underlying absorption profiles (normalised EQE) for the SQ model (grey) and the model TAPC:C_60_ blend (at 6 mol% donor) (green) whose additional Gaussian tail corresponds to the broadening *σ*(*T*) as calculated in Fig. 2b. In both models, *E*_g_ = 1.57 eV of a TAPC:C_60_ blend is used as gap energy. The voltage loss Δ*V*_r_ = $V_{\mathrm{r}}^{\mathrm{SQ}}-V_{\mathrm{r}}^{\mathrm{SQ}+\mathrm{t}}$ is about 0.2 eV at room temperature. **d** Temperature dependence of Δ*V*_r_ for all donor:C_60_ blends based on the broadening *σ*(*T*) from Fig. 2b. **e** Dependence of voltage losses Δ*V*_r_ on the strength of the EPC for the different systems (*T* = 300 K). Dashed grey line indicates the reference Δ*V*_r_ for the microscopic EPC parameters from ab initio simulations and *E*_g_ = $E^{1}_{\mathrm{CT}}$ (i.e. the peak energy of the CT absorption) is taken for the individual system. In **a**, **c** and **d** are the lines guide to the eye.

We evaluate Eq. (4) numerically using the simulated EDOS $D\left( {\frac{E}{\hbar }} \right)$ of the four systems, the numerical AM1.5G spectrum for *ϕ*_Sun_(*E*) and $\Sigma \left( E \right) = fD\left( {\frac{E}{\hbar }} \right)/E$ with *f* the oscillator strength. Fig. 3a shows the temperature-dependent *V*_r_, which depends on the molecular species. For instance, despite higher *E*_CT_ of TAPC:C_60_ as compared with rubrene:C_60_ (Supplementary Table 2), rubrene provides a slightly higher *V*_r_ because of its narrow linewidth. Similarly, the *V*_r_ of m-MTDATA:C_60_ is strongly suppressed by its broad EDOS tail when compared with Spiro-MeO-TPD:C_60_, which has almost the same *E*_CT_, indicating the importance of other molecular parameters for the voltage *V*_r_ apart from *E*_CT_. The agreement with experimentally obtained values, shown in Fig. 3b, is excellent.

To rationalise the influence of molecular parameters on *V*_r_, we compare the cases of finite and infinite absorption tail steepness. The grey curve in the inset of Fig. 3c with infinitely steep absorption tail represents the established Shockley–Queisser (SQ) model^39^ describing an upper limit for the open-circuit voltage $V_{\mathrm{r}}^{\mathrm{SQ}}$ for a given optical gap energy (*E*_g_ = $E_{\mathrm{CT}}^{1}$). While the SQ limit only depends on *E*_g_—a well-established tuning parameter in OSC research to match the solar spectrum^21,40^—we demonstrate that the observed losses depend on additional molecular parameters. Compared with the SQ reference model, we calculate voltage losses due to an additional vibration-induced Gaussian tail below *E*_g_ with a width *σ*(*T*) for Σ(*E*) (green in the inset of Fig. 3c). The solar spectrum is approximated by the black-body radiation *ϕ*_Sun_(*E*) → *ϕ*′_Sun_(*E*,*T*_S_) at the effective temperature of the sun (*T*_S_ = 5800 K), which yields an analytical model for comparison between both cases (see Supplementary Methods). The voltage loss due to the additional low-energy tail is

5 $${\mathrm{\Delta }}V_{\mathrm{r}} = V_{\mathrm{r}}^{{\mathrm{SQ}}} - V_{\mathrm{r}}^{{\mathrm{SQ}} + {\mathrm{t}}},$$

where $V_{\mathrm{r}}^{\mathrm{SQ}+\mathrm{t}}$ is the voltage for the case with finite tail. In Fig. 3c, $V_{\mathrm{r}}^{\mathrm{SQ}+\mathrm{t}}$ is simulated for the TAPC:C_60_ system from the temperature-dependent *σ*(*T*) from Fig. 2b and shows close similarity to the numerical evaluation of Eq. (4) in Fig. 3a, thus indicating the suitability of the “SQ + t” model for further analysis. The tail-induced voltage drop Δ*V*_r_ below the SQ model is temperature dependent but starts to saturate at room temperature (Fig. 3d). This behaviour can be approximated by an analytical expression. In the limit of small or moderate broadening, i.e. *k*_B_*T* ≲ *σ*(*T*) ≪ *E*_g_, we find a simple relation between the radiative losses Δ*V*_r_ and *σ*(*T*), namely

6 $${\mathrm{\Delta }}V_{\mathrm{r}} \approx \frac{{\sigma ^2\left( T \right)}}{{2k_{\mathrm{B}}Te_0}}.$$

This relation describes the behaviour at and below room temperature and closely approaches the numerical results in Fig. 3d. We observe a substantial donor-dependent voltage drop at room temperature (Δ*V*_r_ = 0.12–0.5 V) due to the vibration-induced tail and an increase of these losses upon cooling for all considered donor:C_60_ blends. Therefore, one consequence of zero-point fluctuations is the increase of Δ*V*_r_ upon cooling. We note that at very low temperatures *V*_r_ and Δ*V*_r_ vanish (see Supplementary Methods).

Based on these findings, we elaborate on strategies to reduce these linewidth-induced losses because they can cause a significant reduction of the open-circuit voltage. Since the EPC dominates the temperature-dependent broadening *σ*(*T*) the most intuitive way might be to reduce the coupling to the vibrations. While concrete examples are discussed in the next section, we demonstrate its effect in Fig. 3e by simulating the radiative losses Δ*V*_r_ as a function of a scaling of the total molecular EPC strength for all vibrational modes of donor and acceptor. For the example of m-MTDATA:C_60_, increasing the total EPC strength and keeping all other parameters constant increases Δ*V*_r_ (additional 100 mV for +25% EPC for all vibrational modes), while reducing EPC (−25%) would decrease its voltage losses by about 180 mV at room temperature.

**Fig. 4:**
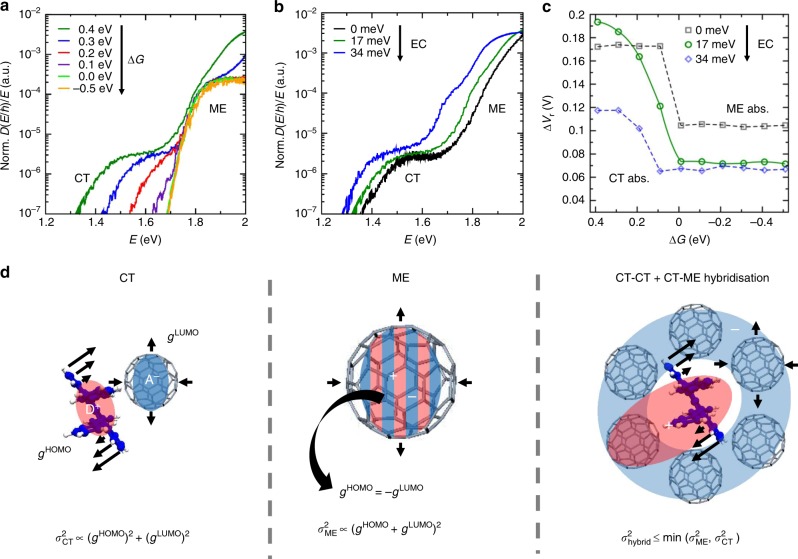
Driving force and EC tuning of the EDOS and schematics of the loss reduction mechanisms. **a** EDOS based on TAPC:C_60_ for varying CT exciton driving energy Δ*G* = *E*_ME,C60_ − $E_{\mathrm{CT}}^{1}$ by increasing the CT energy as a tuning parameter (*E*_ME,C60_ is the energy of the local molecular exciton on C_60_, see Supplementary Methods). Here, we assume a 100 times larger oscillator strength for the molecular excitons. Reducing Δ*G* leads, independently of its purely electronic effect, also to narrower vibration-induced tails. **b** EC dependence of the EDOS of the TAPC:C_60_ blend where *σ*_TI_ varies from 0 to 34 meV. **c** Voltage losses for varying average electronic/excitonic couplings (indicated values characterise a random distribution) plotted as a function of driving energy Δ*G*. Increasing EC between CT excitons and molecular excitons reduces the vibration-induced voltage losses (relative to the SQ limit for *E*_g_ = min [$E_{\mathrm{CT}}^{1}$,*E*_ME_]) substantially. Δ*V*_r_ is minimised for zero driving energy, while finite Δ*G* can be partly compensated by larger EC (blue). **d** Schematic illustration of the different impact of the vibrational coupling for different classes of excitonic states. The EPC-induced broadening for CT states and ME states is expressed by the molecular EPC constants *g*^HOMO^ and *g*^LUMO^ while CT–CT and CT–ME hybridisation modify the EPC-induced broadening. In **c** are the lines guide to the eye.

Additional modifications of other molecular parameters based on the TAPC:C_60_ simulations show that enhanced excitonic coupling (EC) and lower driving energies also reduce the vibration-induced Δ*V*_r_. This is illustrated in Fig. 4a, where we shift the driving energy Δ*G* away from the reference value of Δ*G* = 0.4 eV of the TAPC:C_60_ blend. Lower driving energies shift the CT-peak below the molecular exciton band of the C_60_ host. We observe a significant change in the linewidth while reducing Δ*G*, which results in a significant change in Δ*V*_r_. The losses are reduced from around 0.2 to 0.07 V at Δ*G* ≤ 0 V (see Fig. 4c green circles), which corresponds to the transition from CT to ME exciton absorption at low excitation energies. But why do both type of exciton states have different linewidth?

The reason is the different efficiency of the EPC-induced broadening mechanism for the CT states and the ME states. In Fig. 4d, we illustrate how the broadening of the CT states *σ*_CT_ is composed of the individual EPC contributions from the involved orbitals (given by the coupling constants *g*^HOMO^ and *g*^LUMO^ for an arbitrary vibration mode). The broadening of the CT states is governed by the sum of the squared coupling constants both of which increase the broadening independently of the sign of the couplings. However, for the ME states it is the sum of the coupling constants that is to be squared and that causes the linewidth. This is because the HOMO and the LUMO occupy the same vibrating molecule and opposite charges partly compensate. In contrast, both orbitals in the CT states are typically coupled to the vibrations of the respective molecular species. Therefore, the broadening *σ*_ME_ of the ME states can vanish if the EPC constants cancel each other (i.e. *g*^HOMO^ = −*g*^LUMO^) as illustrated in Fig. 4d. If the overlap of the HOMO and the LUMO on a single molecule is very large, this cancellation effect becomes important. Thus, the EPC-induced broadening is expected to be smaller for ME states than for CT states, which entails a reduction of Δ*V*_r_ with decreasing driving force (Fig. 4c). This compensation effect acts independently of the electronic coupling.

The impact of the EC on the EDOS is further analysed in Fig. 4b by modifying the strength of the electronic coupling while keeping the rotational disorder of randomly oriented C_60_-molecules. We make two observations while changing the EC. Firstly, we identify a slight shift of the EDOS towards lower energies when increasing the EC (see Fig. 4b, blue line)^33^. This shift is due to the stronger hybridisation of different CT states and of CT with ME states due to the increased CT–ME coupling. Secondly, we find an increase in steepness for the low-energy absorption tail with increasing EC for the excitonic states that have dominant CT character. An increasing EC also facilitates the CT–CT hybridisation, which enables the electrons to delocalise over several C_60_ molecules yielding a transition from a Gaussian to an exponential low-energy tail, while at lower EC the Gaussian shape dominates the tail as seen for the EC of 0 meV (black line in Fig. 4b) and 17 meV (green line in Fig. 4b), respectively.

Finally in Fig. 4c, we calculated the resulting radiative voltage losses Δ*V*_r_ (compared with the tail-free SQ limit) for all values of the driving energy Δ*G* and the electronic coupling of standard deviation *σ*_TI_. We find that the radiative losses Δ*V*_r_ are usually smaller at higher EC and that the latter counteracts the EPC and yields a reduced tail broadness *σ*_hybrid_. The effect of the EC is strongest when its energy is comparable with the driving energy—a regime that deserves increased attention in future work.

### Reduction of radiative voltage losses in prototypical systems

**Fig. 5:**
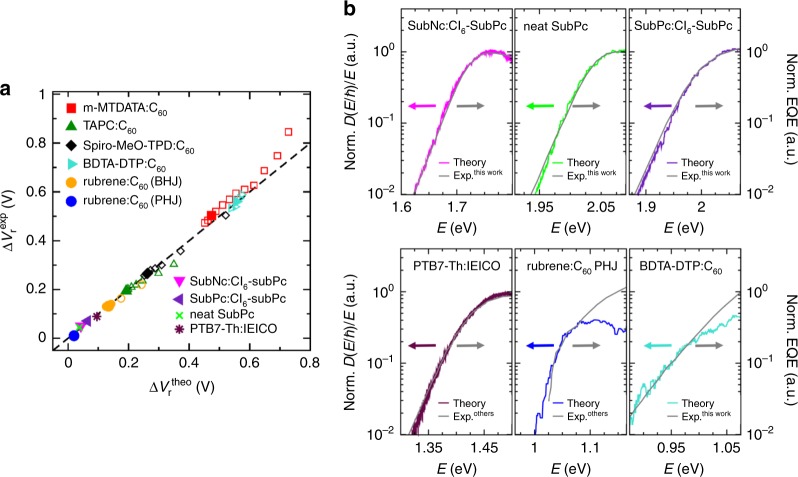
Comparison of theoretical and experimental values for Δ*V*_r_ and sub-gap tails. **a** Comparison of vibration-tail-induced voltage losses Δ*V*_r_ from theory and experiment for all investigated donor–acceptor systems at room temperature (filled symbols) and varying temperatures (open symbols). For rubrene:C_60_, we distinguish bulk heterojunction (BHJ) and planar heterojunction (PHJ) geometries. The dashed line indicates equality between simulated and experimental radiative voltage losses. **b** Simulated spectra and experimental EQE for devices with non-fullerene acceptors and additionally investigated materials. The EQE data for PTB7-Th:IEICO and rubrene:C_60_ are taken from the literature^21, 50^, respectively.

Based on the analysis of the EDOS, we derive guidelines for reducing the linewidth losses that are summarised in Table 1 and for which we extend our study to other classes of systems, e.g. non-fullerene acceptors, and study different heterojunction geometries. Based on these systems, we discuss three strategies to reduce these losses and how they can be implemented. Figure 5a shows an overview that indicates a clear agreement between all simulated and experimental vibration-induced voltage losses and demonstrates material specificity of the theoretical approach.

**Table 1:** Summary of guidelines to reduce vibration-induced radiative voltage losses.

General guideline	Realisation	Examples
(1) Reduction of EPC	∙ Rigidification ∙ Orbital delocalisation ∙ Avoidance of flexible molecular cores by fusing subunits	∙ Oligomers/polymers with rigidly fused molecular cores in contrast to compounds with flexible spacer units ∙ Delocalisation such as in C_60_ or other molecules with increased conjugation length
(2) EPC compensation (*g*^HOMO^ = −*g*^LUMO^)	∙ Molecular orbital shapes for HOMO and LUMO are similar and/or are located on the same part of the molecule (or the entire molecule)	∙ A–D–A compounds with fully conjugated core in contrast to A–D structures on which HOMO and LUMO are more separated
(3) Exciton delocalisation	∙ Increase of the number of transfer integrals ∙ Increase of the number of neighbours ∙ Increase of the values of transfer integrals	∙ Crystalline rubrene film exhibit large transfer integrals at a flat heterojunction as compared with rubrene-C_60_ bulk heterojunction ∙ SubNc and SubPc structures, having many neighbour molecules

Clearly, zero-point fluctuations per se cannot be circumvented. Still, the losses can be reduced even when pure electronic tuning approaches are exhausted^21,22^. The first strategy in this direction is reducing the EPC (cf. Fig. 3e). This can be realised by choosing appropriate material systems with reduced EPCs of low-frequency modes. An example is the rubrene:C_60_ blend with much lower Δ*V*_r_ as compared with the m-MTDATA:C_60_ blend (Fig. 5a). Alternatively one can actively tailor the molecular structure. Here, viable chemical design strategies to reduce the EPC are rigidification and orbital delocalisation^41–43^, for instance by extending the conjugated core (see Table 1 and more detailed discussion in Supplementary Methods). These strategies have already been exploited to tune orbital energies^44,45^.

Secondly, for the class of systems with low driving energy, where the low-energy absorption is dominated by molecular excitons or hybrid states, the EPC strength of electrons and holes on the same molecule should have opposite sign and similar absolute values (for illustration see Fig. 4d). The latter can be realised when the molecular orbital shapes for HOMO and LUMO become similar or located on the same part of the molecule (or the entire molecule). This reduces the effective excitonic EPC and the vibration-induced tail even if HOMO and LUMO on their own are affected by significant vibrational coupling (see Supplementary Methods). Examples are non-fullerene acceptors such as SubNc, SubPc, and Cl_6_-SubPc whose losses are below the best C_60_ based blends (Fig. 5b). Independent evidence for this rule is materialised in the popular class of non-fullerene acceptors based on indacenodithiophene (IDT) or indacenodithienothiophene (IDTT) molecular cores such as IEICO (see Supplementary Fig. 2b)^46,47^, which we take as an example compound from the IDT and IDTT classes. In blends with polymer donors that are adjusted for low driving energy^21^, IEICO is the low-energy absorbing species. We confirm that the EPCs of both electrons and holes are relatively large ($\sigma^{\mathrm{HOMO}}_{\mathrm{IEICO}}$ = 58.5 meV and $\sigma^{\mathrm{LUMO}}_{\mathrm{IEICO}}$  = 55.6 meV) but similar in size, while the resulting excitonic EPC is below both individual contributions. We predict a small Gaussian linewidth ($\sigma^{\mathrm{ME}}_{\mathrm{IEICO}}$ = 55.4 meV). The narrow absorption tail^21^ (Fig. 5b) is therefore a consequence of the HOMO and LUMO being delocalised over the same part of the molecule or the entire molecule, which can be induced by a symmetric acceptor–donor–acceptor (A–D–A) type molecular structure. It manifests in a reduced vibration-induced radiative voltage loss of 95 mV. In general, acceptors of A–D–A type should avoid a separation of the frontier electronic states (e.g. that the LUMO is located on the acceptor part and the HOMO on the donor part). We believe that also the recent record performances of PM6:Y6 blends^48,49^ would be likely unachievable if the non-fullerene acceptor Y6 did not follow the guidelines outlined in Table 1.

Finally, the third strategy is to increase the EC by means of the number of transfer integrals (e.g. number of neighbours) and their size. Transfer integrals couple different CT states and also CT excitons with molecular excitons. For example, in neat SubNc, CT states would have even narrower tails (19 meV Urbach energy) as compared with molecular excitons despite somewhat larger effective EPCs. The reason is that the larger number of transfer integrals that couple such CT states overcompensates the disadvantageous EPC increase. Another example for this strategy has been realised experimentally with a planar heterojunction interface of rubrene:C_60_^50^. As compared with bulk heterojunctions of the same compounds, structural order in the donor domain facilitates large hole transfer integrals (134 meV) between adjacent rubrene molecules^51^. This coupling connects different rubrene:C_60_ CT states, which results in an Urbach energy of 14 meV and a voltage loss Δ*V*_r_ as low as 20 mV, which is much smaller than the 130 mV for the BHJ with the same materials (Fig. 5a) and agrees well with the literature^50^.

## Discussion

We conclude that strong electron–electron interaction and EPC, which are caused by the molecular structure of the organic semiconductors, determine the properties of the manifold of low-energy excitons that includes molecular excitons, CT excitons and hybrids. The molecular vibrations of low-frequency modes (including zero-point effects) impact the absorption properties of OSCs and hence their device parameters. The developed theory can be used to describe their sub-gap absorption. The proposed strategies to minimise vibration-induced voltage losses (of up to several hundred meV, depending on the system), i.e. reduction of EPC, EPC compensation and exciton delocalisation, are supported by the excellent agreement between theory and experiment for many systems in Fig. 5a. These strategies should therefore be considered in future research to mitigate the effect of the molecular vibrations and to lower radiative voltage losses down to the best inorganic semiconductors.

## Methods

### Theoretical approach

Our approach to describe excitons and vibrations in the studied systems is based on a general form of the Hamiltonian ***H*** = *H*_el_ + *H*_el−el_ + *H*_el−ph_ + *H*_ph_, where $H_{{\mathrm{el}}} = \mathop {\sum }\nolimits_{vMN} \varepsilon _{vMN}a_{vM}^\dagger a_{vN}$ is the electronic (one-body) Hamiltonian with the indices *M* (*N*) running over sites and ***v*** over orbitals, i.e. HOMO- and LUMO-derived states, $H_{{\mathrm{el}} - {\mathrm{el}}} = \mathop {\sum }\nolimits_{KLMN} V_{KLMN}a_K^\dagger a_L^\dagger a_Ma_N$ is the Coulomb interaction term, $H_{{\mathrm{ph}}} = \mathop {\sum }\nolimits_\lambda \hbar \omega _\lambda \left( {b_{M\lambda }^\dagger b_{M\lambda } + \frac{1}{2}} \right)$ describes the molecular vibrations and $H_{{\mathrm{el}} - {\mathrm{ph}}} = \mathop {\sum }\nolimits_{v\lambda M} g_{vMM}^\lambda \hbar \omega _\lambda \left( {b_{M\lambda }^\dagger + b_{M\lambda }} \right)a_{vM}^\dagger a_{vM}$ their local EPC with electrons. The coupling constant $g_{vMM}^\lambda$ describes the changes in the site energy (site *M*) due to mode *λ*^52^. The different molecular species, donor and acceptor molecules, are encoded in the Hamiltonian with their local orbital energies *ε*_*vMN*_, which are given by the molecular ionisation energies and electron affinities in bulk phase. The material parameters, which include also the vibrations and their coupling, are obtained by ab initio calculations (see Supplementary Methods for more details on the modelling). The definition of the exciton density of states in Eq. (2) of the main text describes the limit that in the initial state no excitons or charges are present and the indices to the electron creation $\left( {a_l^\dagger } \right)$ and annihilation (*a*_*h*_) operators run over unoccupied and occupied set of states, respectively. Numerically, the EDOS is obtained from the Hamiltonian in electron–hole tensor space with a localised electron–hole basis. System sizes of about 40,000 molecules are employed in the EDOS simulations. We calculate the spectral function with a Lanczos approach^31^ and continued fraction expansion, which are well-established techniques^53^ that are applied here to the large sets of exciton states. Specific details are discussed in Supplementary Methods.

### Device preparation

The photovoltaic devices are thermally evaporated at ultra-high vacuum (base pressure lower than 10^−7^ mbar) onto a glass substrate with a pre-structured ITO contact (Thin Film Devices, USA). Two nanometers of MoO_3_ is deposited to adjust the ITO work function. The active layer comprises 50 nm of C_60_ (CreaPhys GmbH, Germany) doped with 6 mol% of each donor molecule. Afterwards, 8 nm of Bathophenanthroline (BPhen, abcr GmbH, Germany), used as electron contact, is evaporated and finished with 100 nm of Al. The donor materials rubrene and TAPC were supplied by Sensient, m-MTDATA and Spiro-MeO-TPD by Lumtec and BDTA-DTP by the Georgia Institute of Technology. Before processing, the organic materials were purified two to three times by sublimation. The device is defined by the geometrical overlap of the bottom and the top contact with an active area of 6.44 mm^2^. To avoid exposure to ambient conditions, the organic part of the device is covered by a small glass substrate, glued on top. The neat SubNc device is comprised ITO (90 nm), MoO3 (3 nm), SubNc (12 nm), BPhen (8 nm) and Ag (100 nm). The neat SubPc device is comprised ITO (90 nm), BPAPF:NDP9 (10 wt%, 30 nm), BPAPF (5 nm), SubPc (20 nm), BPhen (8 nm) and Ag (100 nm). The SubPc/Cl_6_-SubPc is comprised ITO (90 nm), BPAPF:NDP9 (10 wt%, 30 nm), BPAPF (5 nm), SubPc (21 nm), Cl_6_-SubPc (21 nm), BPhen (8 nm) and Ag (100 nm). All layers are sequentially deposited apart from the p-doped BPAPF:NDP9 layer. BPAPF, SubNc, SubPc and Cl_6_-SubPc were purchased from Lumtec (Taiwan), while NDP9 is a p-dopant purchased from Novaled (Germany). Ag and Al were purchased from Kurt. J. Lesker company and MoO_3_ from Sigma Aldrich.

### Sensitive EQE measurements

The light of a quartz halogen lamp (50 W) is chopped at 140 Hz and coupled into a monochromator (Cornerstone 260 1/m, Newport). The resulting monochromatic light is focused onto the device that is mounted on a liquid nitrogen cryostat (Optistat DN, Oxford Instruments, UK) with programmable temperature controller. To ensure a constant temperature at the set value, the device itself was used as a thermometer by measuring the temperature sensitive open-circuit voltage, induced by the monochromatic illumination. After ca. 20 min, the measured open-circuit voltage saturates and the set temperature has been reached in the cryostat. The measurement was started after 10 min additional settling time. The device’s short-circuit current is fed to a current pre-amplifier before it is analysed with a lock-in amplifier (7280 DSP, Signal Recovery, Oak Ridge, USA). The time constant of the lock-in amplifier was chosen to be 1 s and the amplification of the pre-amplifier was increased to resolve low photocurrents. The EQE is determined by dividing the photocurrent by the flux of incoming photons, which was obtained with a calibrated silicon and/or indium-gallium-arsenide photodiode.

## Supplementary information

Supplementary Information

## Data Availability

All the data supporting the findings of this study are available within the article, its Supplementary Material files or from the corresponding authors upon reasonable request.

## Code availability

The custom codes used for the simulations are available from the corresponding author upon reasonable request.
