# Author Correction: Molecular vibrations reduce the maximum achievable photovoltage in organic solar cells

**DOI:** 10.1038/s41467-020-15790-z

**Published:** 2020-04-22

**Authors:** Michel Panhans, Sebastian Hutsch, Johannes Benduhn, Karl Sebastian Schellhammer, Vasileios C. Nikolis, Tim Vangerven, Koen Vandewal, Frank Ortmann

**Affiliations:** 1 Center for Advancing Electronics Dresden, Technische Universität Dresden, 01062 Dresden, Germany; 2 Dresden Integrated Center for Applied Physics and Photonic Materials (IAPP) and Institute for Applied Physics, Technische Universität Dresden, Nöthnitzer Str. 61, 01187 Dresden, Germany; 3 Institute for Materials Research (IMO-IMOMEC), Hasselt University, Wetenschapspark 1, 3590 Diepenbeek, Belgium

**Keywords:** Excited states, Solar cells, Condensed-matter physics

Correction to: *Nature Communications* 10.1038/s41467-020-15215-x, published online 20 March 2020.

The original version of this Article contained two typos in the last three lines of the figure caption of Fig. 1, which incorrectly read ‘**c** EDOS including EC between molecules…’ and ‘Orange lines indicate analytical energies from **b**’. The correct version states ‘**e** EDOS including EC between molecules…’ and ‘Orange lines indicate analytical energies from **c**’, respectively.

This has now been corrected in both the PDF and HTML versions of the Article.

