# Supplementary material for: Molecular vibrations reduce the maximum achievable photovoltage in organic solar cells
Source: Nat Commun. 2020 Mar 20;11:1488. doi: 10.1038/s41467-020-15215-x (PMC7083957; doi:10.1038/s41467-020-15215-x)
Supplement: Supplementary file 1 — Supplementary Information [file 41467_2020_15215_MOESM1_ESM.pdf]

Supplementary Material for

Molecular vibrations reduce the maximum achievable photovoltage in organic solar cells

By

Michel Panhans, Sebastian Hutsch, Johannes Benduhn, Karl Sebastian Schellhammer, Vasileios C. Nikolis, Tim Vangerven, Koen Vandewal, Frank Ortmann

Correspondence to: frank.ortmann@tu-dresden.de

Supplementary Figures

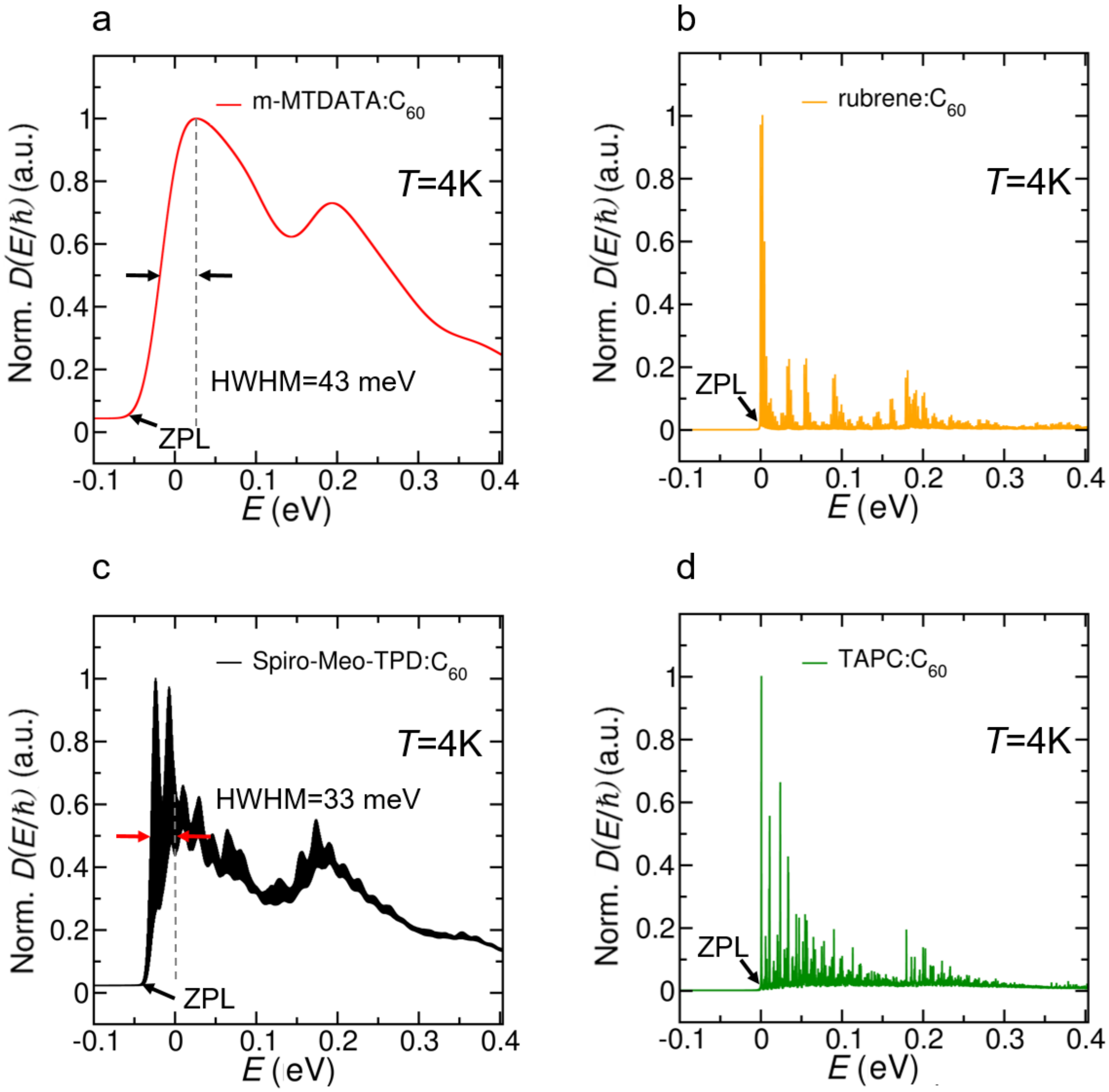

Supplementary Figure 1: Normalised EDOS of the single-state model. Spectra are calculated with Supplementary Equation 67 for a single state at 4 K where the energy scale and the value of the $D\left(\frac{E}{\hbar}\right)$ are normalised for better comparison. The half width at half maximum (HWHM) and the position of the zero-phonon line (electronic transition for the vibrational ground states of all modes) are indicated.-

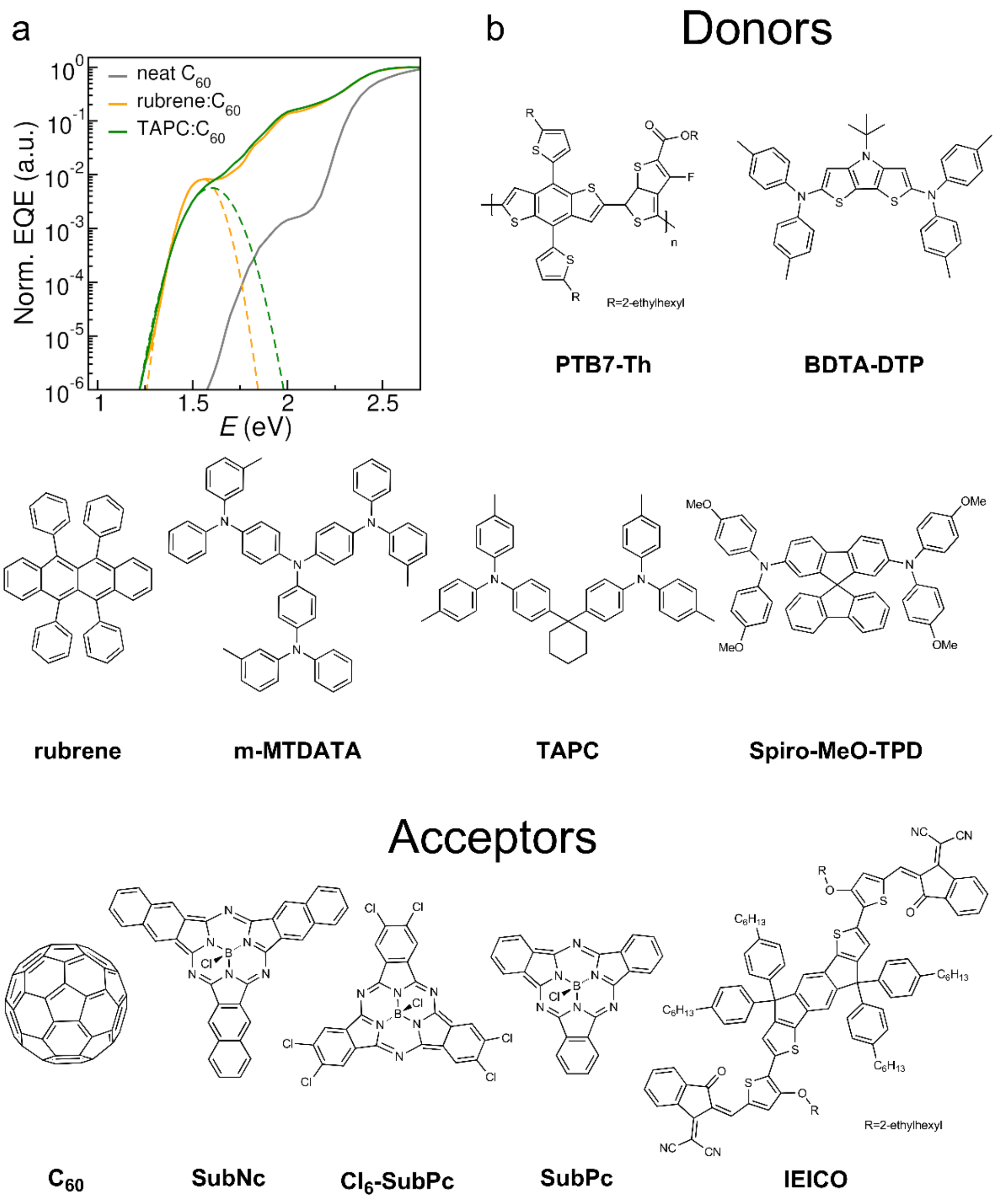

Supplementary Figure 2: Experimental EQE spectra at 300 K with low-energy CT tail. (a) Measured EQE spectra of representative donor:C_{60} blends, with rubrene and TAPC being the donor molecules, respectively. The lineshapes are fitted with a Gaussian function at the low-energy tail (shown as dashed lines). Extracted linewidth are σ_{exp} =69 meV (rubrene) and σ_{exp} =91 meV (TAPC). We emphasise that the CT absorption features of both blends only occur in presence of both donor and acceptor molecules and scale proportional to the donor:C_{60} ratio[1]. Pristine C_{60} data (grey solid line) is measured for

reference, demonstrating the absence of a low-energy absorption of donor-acceptor type. The optical gaps of the donors are even larger. (b) Molecular structures of the compounds studied in this work.

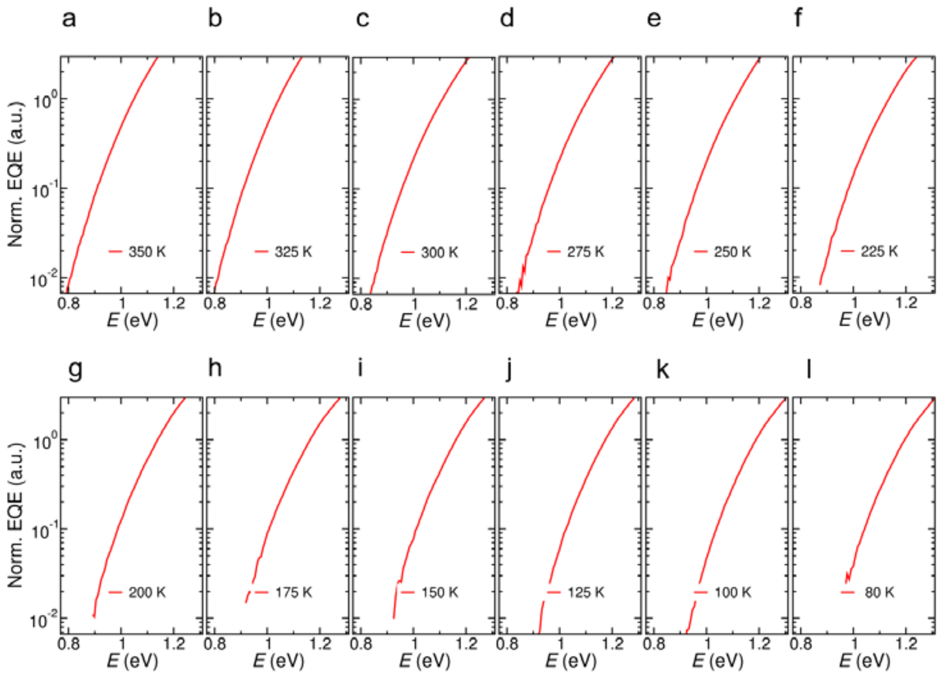

Supplementary Figure 3: Experimental low-energy EQE spectra for m-MTDATA:C_{60} for all measured temperatures.

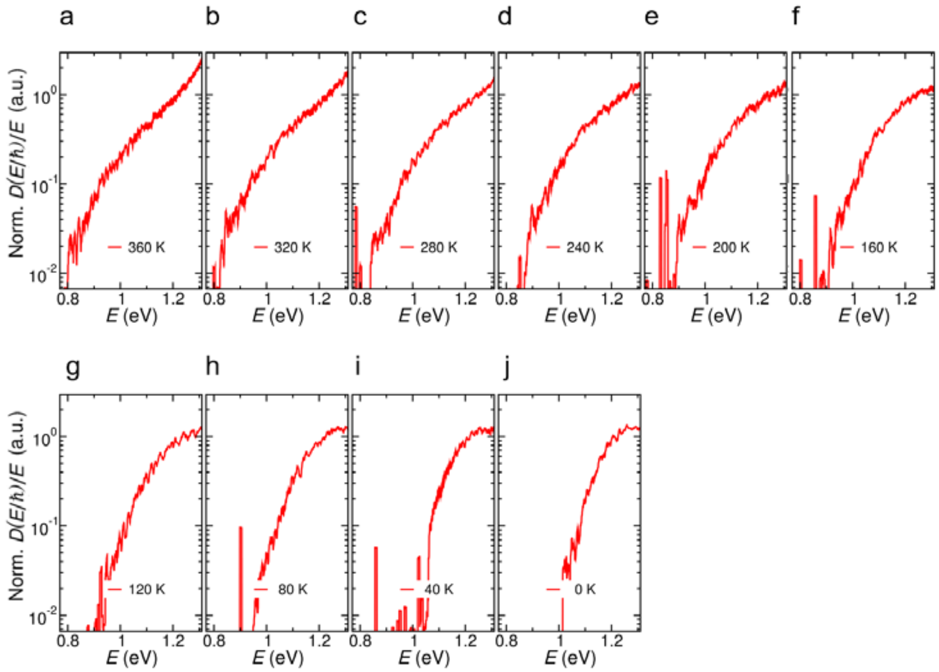

Supplementary Figure 4: Normalised low-energy EDOS divided by energy for m-MTDATA:C_{60} at all simulated temperatures.

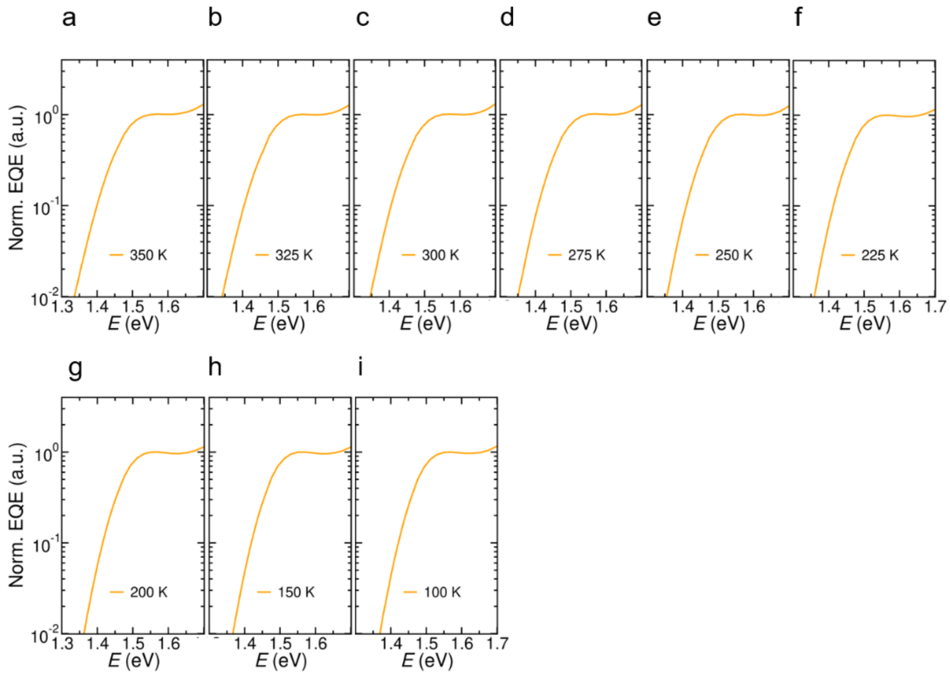

Supplementary Figure 5: Experimental low-energy EQE spectra for rubrene:C_{60} for all measured temperatures.

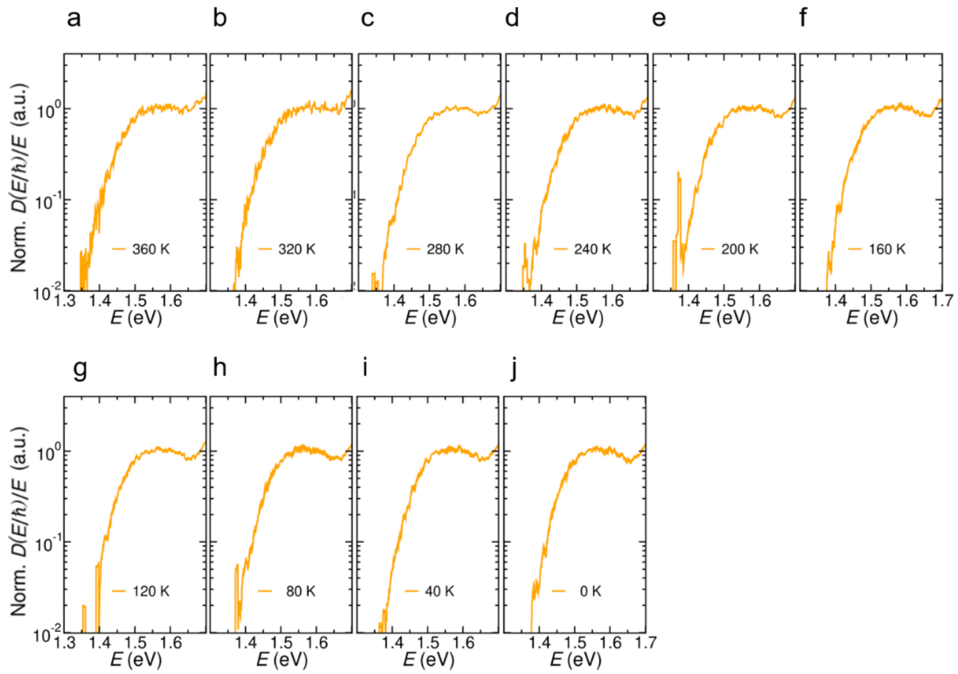

Supplementary Figure 6: Normalised low-energy EDOS divided by energy for rubrene:C_{60} at all simulated temperatures.

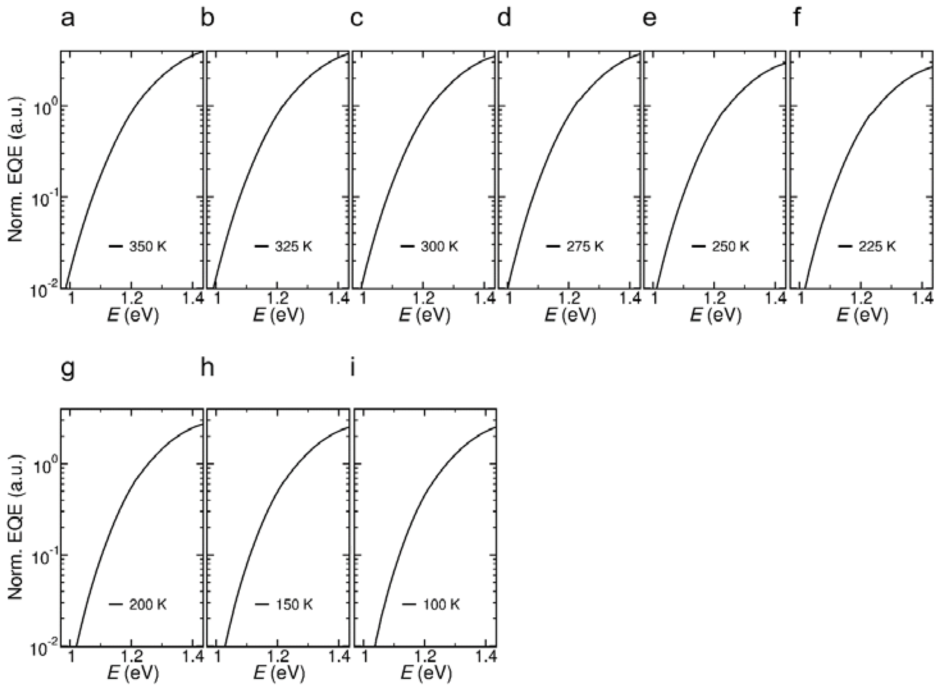

Supplementary Figure 7: Experimental low-energy EQE spectra for Spiro-MeO-TPD:C_{60} for all measured temperatures.

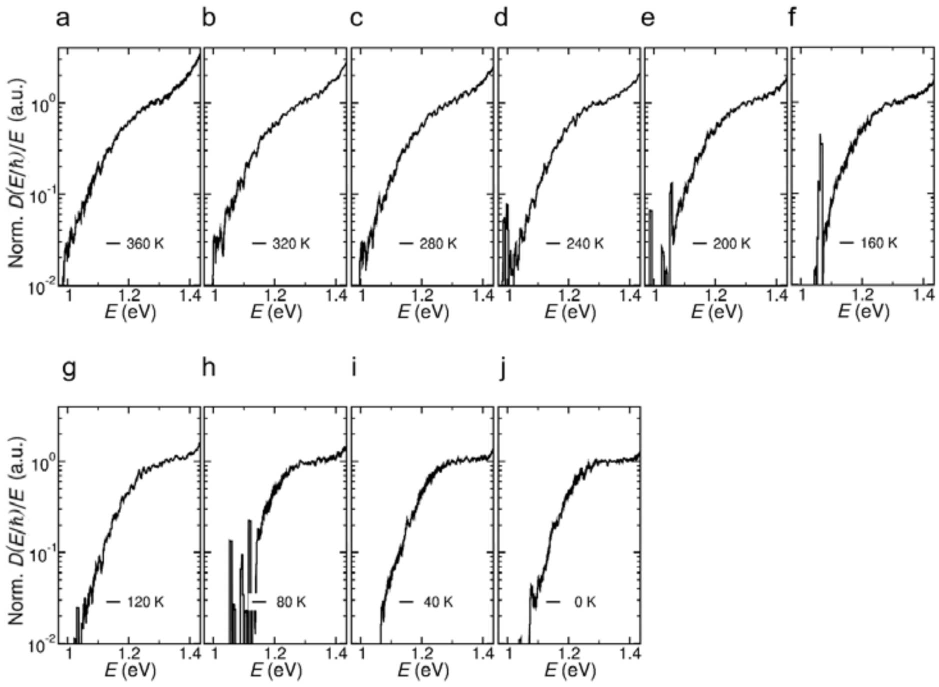

Supplementary Figure 8: Normalised low-energy EDOS divided by energy for Spiro-MeO-TPD:C_{60} at all simulated temperatures.

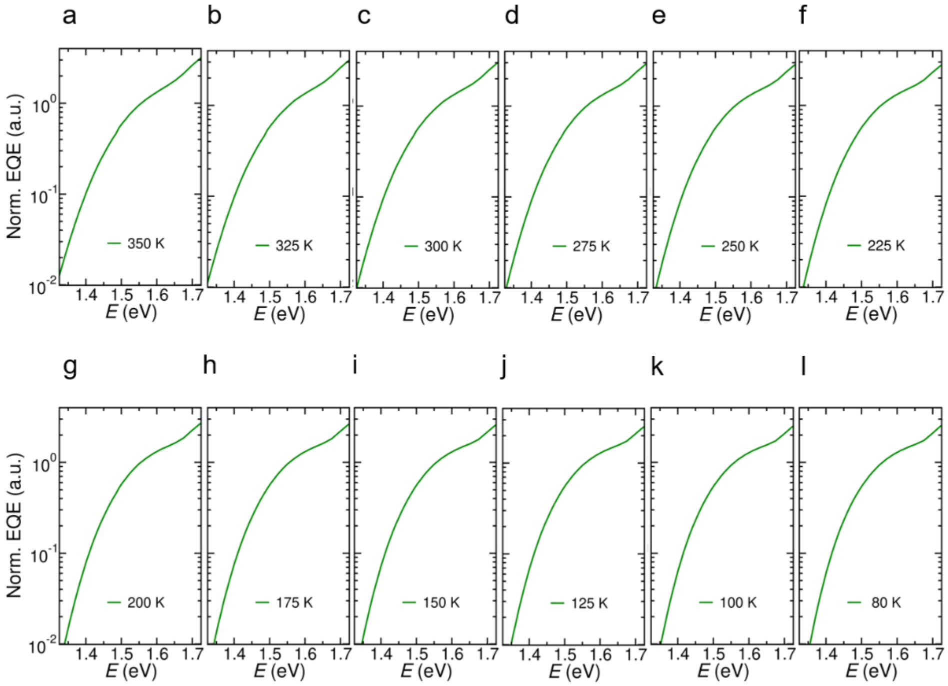

Supplementary Figure 9: Experimental low-energy EQE spectra for TAPC:C_{60} for all measured temperatures.

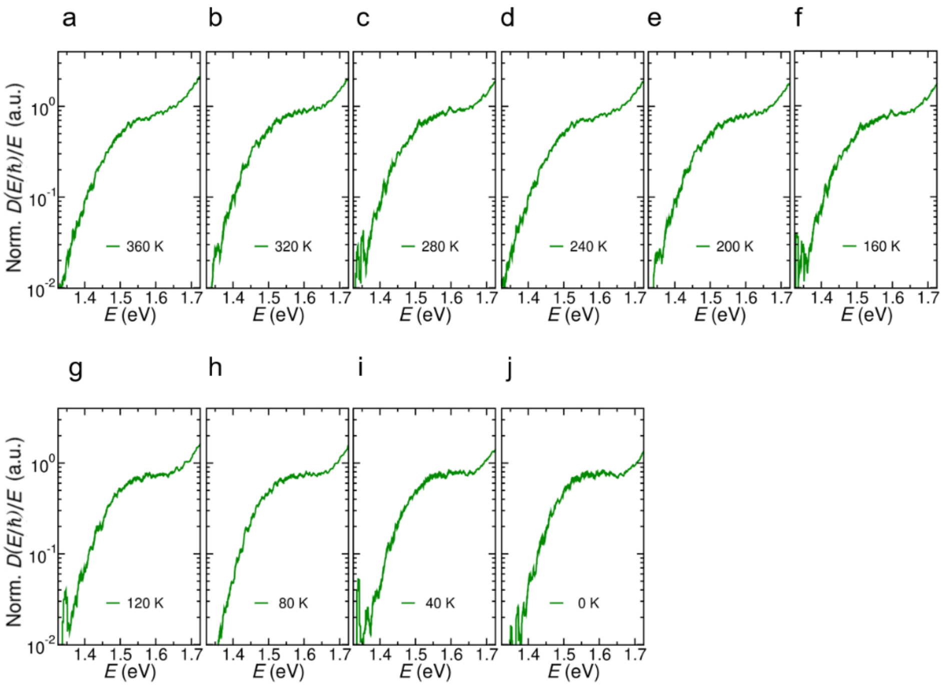

Supplementary Figure 10: Normalised low-energy EDOS divided by energy for TAPC:C_{60} at all simulated temperatures.

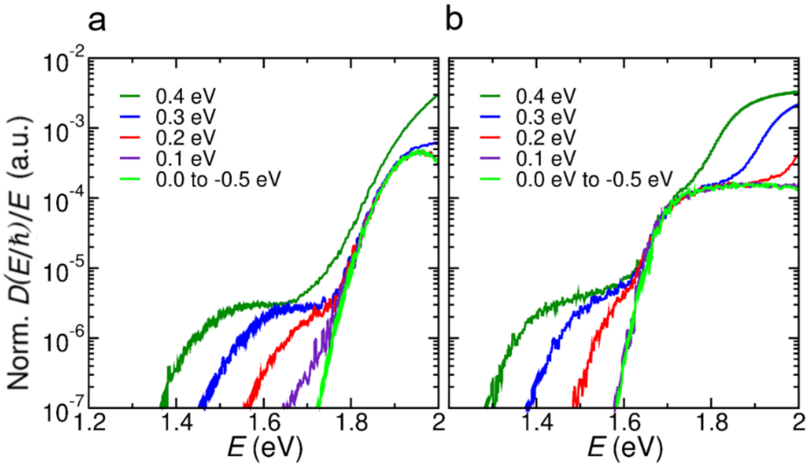

Supplementary Figure 11: Normalised low-energy EDOS divided by energy for TAPC:C_{60} at varying driving energy ΔG and EC. (a) EC is set to zero and ΔG varies from 0.4 eV to negative values of -0.5 eV where the EDOS from driving energies below 0.0 eV have the same lineshape since no CT feature is present and only intrinsic C_{60} absorption is observed. (b) EC is set to σ_{TI} = 34 meV that corresponds to twice the C_{60} value based on the orientational disorder of the C_{60} molecules. Here curves for zero and negative values have the same form and thus are not displayed here for simplicity.

Supplementary Tables

Material parameters	rubrene	TAPC	Spiro-MeO-TPD	m-MTDATA
Experimental $IP_{\mathrm{D}}(\mathrm{D})$ (eV)	---	5.85[2]	5.5[3]	5.5[4]
Dielectric constant	---	3.0[5]	3.0[6]	2.7[7]
Radius R_{cyl} (Å)	---	7.29	6.87	6.26
Polarisation $P_{\mathrm{D}}(\mathrm{D})$ (eV)	---	0.48	0.51	0.53
Gas phase $IP_{\mathrm{gas}}(\mathrm{D})$ (eV)	6.52[8]	6.33	6.01	6.03

Supplementary Table 1: Thin film and gas-phase ionisation potential of various donor molecules.

Material parameter	rubrene	TAPC	Spiro-MeO-TPD	m-MTDATA
Δ_{MD} (eV)	---	---	-0.46	-0.52
$E_{\mathrm{CT}}^{\mathrm{theo}}$ (eV)	1.54	1.57	1.34	1.31
$E_{\mathrm{CT,peak}}^{\mathrm{exp}}$ (eV)[9]	1.54	1.61	1.36	1.36

Supplementary Table 2: Theoretical CT-peak energies obtained from Supplementary Equation 34 and/or in combination with the MD+TD-DFT approach (see text) in comparison to experimental CT-peak energies.

Material parameter	rubrene	TAPC	Spiro-MeO-TPD	m-MTDATA
Δ_0 (eV)	1.89	1.31	1.70	1.74
P_{CT} (eV)	1.29	0.72	1.07	0.99
V_{CT} (eV)	0.60	0.59	0.46	0.44
$IP_{\mathrm{A}}(\mathrm{D})$ (eV)	5.64	5.66	5.30	5.25

Supplementary Table 3: Gas phase electron-hole interaction energy Δ_0, polarisation correction P_{CT}, polarisation corrected bulk electron-hole attraction V_{CT} and MD corrected donor ionisation potential. All values are calculated in bulk C_{60}.

System	$E_{\mathrm{g,exp}}$ (eV)	σ_{loc} (meV)	σ_{TI} (meV)	$\varepsilon_{jj'}^{\mathrm{max}}$ (meV)
SubNc:Cl_6-SubPc	1.75	44 (SubNc)<br>47 (Cl_6-SubPc)	0*	40 (SubNc, LUMO)
neat SubPc	2.08	48	0*	74 (SubPc, HOMO)
SubPc:Cl_6-SubPc	2.08	48 (SubPc)<br>47 (Cl_6-SubPc)	0*	74 (SubPc, HOMO)
IEICO:PTB7-Th	1.46[10]	55	0*	0*
rubrene:C_{60} FHJ	1.07[11]	67	0* (rubrene)<br>17.0 (C_{60})	134[12] (rubrene, HOMO)
BDTA-DTP:C_{60}	1.31[9]	174	17.0 (C_{60})	0*

Supplementary Table 4: Material properties used in the EDOS simulations for different donor:acceptor systems. σ_{loc} is the Gaussian standard deviation from Supplementary Equation 18 depending on the exciton species (ME–exciton or CT–excitons) that cause the absorption tail. σ_{TI} denotes the strength of the orientational disorder (C_{60} only). $\varepsilon_{jj'}^{\mathrm{max}}$ is the maximum transfer integral of the molecular orbital that defines the largest EC in the system where the orbital (HOMO or LUMO) and the molecular species are indicated. Values without references have been obtained experimentally/theoretically during this work. Values with (*) have been set to zero in the calculations.

EPC induced broadening	SubNc	SubPc	Cl_6-SubPc
σ^{HOMO} (meV)	38.8	48.2	51.5
σ^{LUMO} (meV)	48.9	59.0	60.7
$\sqrt{(\sigma^{\mathrm{HOMO}})^2 + (\sigma^{\mathrm{LUMO}})^2}$ (meV)	62.4	70.5	79.6
σ^{ME} (meV)	44.4	48.3	46.9
$\Delta\sigma$ (meV)	18.0	22.2	32.7
EPC compensation (%)	28.9	31.5	41.1

Supplementary Table 5: Comparison of EPC induced broadening of individual HOMO and LUMO contributions with the broadening of molecular excitons for the non-fullerene acceptors SubNc, SubPc and Cl_6-SubPc at 300K. The EPC compensation is the absolute difference $\Delta\sigma$ normalised to the broadening of the individual contributions.

Supplementary Methods

SM.1 Derivation of the theoretical approach

Hamiltonian – The electronic and vibrational properties of the studied systems as well as their interactions can be described by

$$H = H_{\mathrm{el}} + H_{\mathrm{el-el}} + H_{\mathrm{el-ph}} + H_{\mathrm{ph}}, \tag{1}$$

where

$$H_{\mathrm{el}} = \sum_{\nu MN} \varepsilon_{\nu MN} a_{\nu M}^{\dagger} a_{\nu N} \tag{2}$$

is the effective electronic (one-body) Hamiltonian with basis indices running over sites M (N) and over orbitals ν centred on the respective site, i.e. in our case the HOMO- and LUMO-derived states. $H_{\mathrm{el-el}}$ is the (screened) interaction term describing the electron-hole coupling. The different molecular species, donor and acceptor molecules, are characterised by their molecular ionisation energies and electron affinities in bulk phase, i.e. the local orbital energies $\varepsilon_{\nu MN}$ in Supplementary Equation 2. The material parameters in Supplementary Equation 1 are obtained by *ab initio* calculations (see SM.3 and SM.4).

The phonon part of the Hamiltonian is described by harmonic oscillators $H_{\mathrm{ph}} = \sum_{\lambda M} \hbar\omega_{\lambda} \left(b_{M\lambda}^{\dagger} b_{M\lambda} + \frac{1}{2} \right)$ including low-frequency and high-frequency modes with their intramolecular electron-phonon coupling (EPC) $H_{\mathrm{el-ph}}$ according to

$$H_{\mathrm{el-ph}} = \sum_{\nu\lambda M} g_{\nu MM}^{\lambda} \hbar\omega_{\lambda} \left(b_{M\lambda}^{\dagger} + b_{M\lambda} \right) a_{\nu M}^{\dagger} a_{\nu M}. \tag{3}$$

Here, the orbital indexing follows the notation in Supplementary Equation 2 and λ denotes the vibration mode with mode energy $\hbar\omega_{\lambda}$ and dimensionless coupling constants $g_{\nu MM}^{\lambda}$. The material parameters for the vibrational frequencies $\hbar\omega_{\lambda}$ and the EPC constants $g_{\nu MM}^{\lambda}$ of the intra-molecular modes are discussed in section SM.3 of the Supplementary Methods.

Henceforth we use the simplified notation $a_{\nu M}^{\dagger} \to a_{l}^{\dagger}$ for indexes when ν refers to LUMO-type orbitals and $a_{\nu M}^{\dagger} \to a_{h}^{\dagger}$ when ν refers to HOMO-type orbitals (for all molecular species), because the excitations of interest are the energetically lowest ones including Frenkel-type molecular excitons (ME) and charge-transfer (CT) excitons. We restrict our model to those excitations. More orbitals can be added

straightforwardly if necessary. The electron creation ($a_l^\dagger, a_h^\dagger$) and annihilation (a_l, a_h) operators then run over *the set of all* unoccupied (LUMO) and occupied (HOMO) states, respectively.

The electron-electron interaction $H_{\text{el-el}} = \sum_{hl} V_{lhhl}(\mathbf{R}_l - \mathbf{R}_h)\, a_l^\dagger a_h a_h^\dagger a_l + \sum_{lhl'h'} V_{lhl'h'}\, a_l^\dagger a_h a_{h'}^\dagger a_{l'}$ between a LUMO-type electron and a HOMO-type hole is considered in a form that conserves the orbital occupation number $n_{l(h)} = \sum_{l(h)} a_{l(h)}^\dagger a_{l(h)}$, thus excluding effects of intrinsic exciton annihilation or creation. Furthermore, we consider the direct electron-electron interaction with respect to the inter-molecular distance $\boldsymbol{R}_l - \boldsymbol{R}_h$ through

$$V_{lhhl}(\boldsymbol{R}_l - \boldsymbol{R}_h) = \begin{cases} -V_{\text{CT}} \cdot \frac{|\boldsymbol{R}_{\text{min}}|}{|\boldsymbol{R}_l - \boldsymbol{R}_h|} & \forall\, \boldsymbol{R}_l \neq \boldsymbol{R}_h \\ -V_{\text{ME}} & \boldsymbol{R}_l = \boldsymbol{R}_h \end{cases} \tag{4}$$

and the exchange-type terms

$$V_{lhl'h'} = \begin{cases} t_{lhl'h'}^{\text{ME}} & \forall h, l \text{ with } \boldsymbol{R}_l = \boldsymbol{R}_h \text{ and } \forall h', l' \text{ with } \boldsymbol{R}_{l'} = \boldsymbol{R}_{h'} \text{ that are next neighbors of } h, l \\ 0 & \text{otherwise} \end{cases}. \tag{5}$$

$\boldsymbol{R}_{\text{min}}$ is the distance between two next-neighbor molecules. V_{CT} is the electron-hole binding energy of two neighboring molecules that is calculated according to section SM.4 below. V_{ME} is the binding energy of the molecular exciton with a value discussed in section SM.2 below. Finally, $t_{lhl'h'}^{\text{ME}}$ denotes excitonic transfer integrals, which are typically dominated by the dipole-dipole interaction of transition dipoles at neighboring molecules. This contribution describes the delocalisation of Frenkel excitons and is assumed to be at most on the order of the electronic transfer integrals or below. Since these parameters depend on wave function overlap, analogous quantities for CT states are orders of magnitude smaller and can be neglected.

Exciton DOS – We turn to the description of the exciton DOS (EDOS) as defined in Equation 2 of the main text. For its calculation, we specify a subset of initial electron-phonon states for exciton absorption. For these initial states we consider product states $|\psi^{(0)}\rangle|\phi_{\text{ph}}^i\rangle$, where the electron part is denoted by $|\psi^{(0)}\rangle$ and is the fully occupied set of HOMO-type orbitals (corresponding to the charge neutral configuration without thermal exciton occupation). For the phonon part, we use states $|\phi_{\text{ph}}^i\rangle$ that diagonalise H in the neutral electronic ground state:

$$H|\psi^{(0)}\rangle|\phi_{\text{ph}}^i\rangle = \left(E_{\text{el}}^{(0)} + H_{\text{ph}}\right)|\psi^{(0)}\rangle|\phi_{\text{ph}}^i\rangle = \left(E_{\text{el}}^{(0)} + E_{\text{ph}}^{(i)}\right)|\psi^{(0)}\rangle|\phi_{\text{ph}}^i\rangle. \tag{6}$$

As a result, the EDOS in Equation 2 reduces to

$$D(\omega) = \sum_{hl} \sum_i \langle \phi_{\rm ph}^i | \langle \psi^{(0)} | \rho_{\rm ph} a_h^\dagger a_l \delta \left(\hbar\omega + E_{\rm el}^{(0)} + E_{\rm ph}^{(i)} - H \right) a_l^\dagger a_h | \psi^{(0)} \rangle | \phi_{\rm ph}^i \rangle. \qquad (7)$$

To simplify the analysis regarding high-frequency vibrations, we split the set of modes into a group of low-frequency vibrations $H_{\rm ph}^{\rm lf}$ that may be thermally occupied and a group of high-frequency vibrations $H_{\rm ph}^{\rm hf}$ whose Hilbert space is restricted to the respective vibrational ground state denoted as $|0_{\rm ph}^{\rm hf}\rangle$. As a result, the density operator $\rho_{\rm ph}$ of the initial vibrational state reads

$$\rho_{\rm ph} := |0_{\rm ph}^{\rm hf}\rangle\langle 0_{\rm ph}^{\rm hf}| \otimes \rho_{\rm ph}^{\rm lf} \qquad (8)$$

$$\rho_{\rm ph}^{\rm lf} = \frac{e^{-\beta H_{\rm ph}^{\rm lf}}}{Z_{H_{\rm ph}^{\rm lf}}} \qquad (9)$$

with $Z_{H_{\rm ph}^{\rm lf}}$ the corresponding partition function and $\beta = (k_{\rm B}T)^{-1}$. A more complex model for high-frequency modes is not necessary here.

Inserting $\rho_{\rm ph}$ into the EDOS yields

$$D(\omega) = \sum_{hl} \sum_i \langle \phi_{\rm ph,lf}^{\rm lf,\it i} |_{\rm ev} \langle 0 | \rho_{\rm ph}^{\rm lf} a_h^\dagger a_l \delta \left(\hbar\omega + E_{\rm el}^{(0)} + E_{\rm ph}^{{\rm lf},(i)} + E_{\rm ph}^{{\rm hf},(0)} - H \right) a_l^\dagger a_h | 0 \rangle_{\rm ev} | \phi_{\rm ph}^{\rm lf,\it i} \rangle \qquad (10)$$

with the electro-vibronic ground state $|0\rangle_{\rm ev} = |\psi^{(0)}\rangle |0_{\rm ph}^{\rm hf}\rangle$ and the remaining trace ${\rm Tr}_{\rm ph}^{\rm lf}[\ldots] = \sum_i \langle \phi_{\rm ph}^{\rm lf,\it i} | \ldots | \phi_{\rm ph}^{\rm lf,\it i} \rangle$ over the states describing low-frequency vibrations $|\phi_{\rm ph}^{\rm lf,\it i}\rangle$ which can be written as

$$D(\omega) = -\frac{i}{\hbar} \int_{-\infty}^{\infty} {\rm d}t \, e^{i\omega t} \sum_{hl} {\rm Tr}_{\rm ph}^{\rm lf} \left[\langle 0 |_{\rm ev} \rho_{\rm ph}^{\rm lf} e^{\frac{it\left(E_{\rm el}^{(0)} + H_{\rm ph}\right)}{\hbar}} a_h^\dagger a_l e^{-\frac{itH}{\hbar}} a_l^\dagger a_h | 0 \rangle_{\rm ev} \right]. \qquad (11)$$

We can approximate the time evolution operator $e^{\frac{-itH}{\hbar}} \approx e^{\frac{-it\left(H - H_{\rm ph}^{\rm lf}\right)}{\hbar}} e^{\frac{-it\left(H_{\rm ph}^{\rm lf}\right)}{\hbar}}$, which holds if the corresponding commutator vanishes or if the correction terms are small. This is realised when the intrinsic time scales of $H - H_{\rm ph}^{\rm lf}$ are much smaller than the time scale of $H_{\rm ph}^{\rm lf}$. We obtain

$$D(\omega) = -\frac{i}{\hbar} \int_{-\infty}^{\infty} {\rm d}t \, e^{\frac{it\left(\hbar\omega + E_{\rm el}^{(0)} + E_{\rm ph}^{{\rm hf},(0)}\right)}{\hbar}} \sum_{hl} {\rm Tr}_{\rm ph}^{\rm slow} \left[\langle 0 |_{\rm ev} \rho_{\rm ph}^{\rm lf} a_h^\dagger a_l e^{\frac{-it\left(H - H_{\rm ph}^{\rm lf}\right)}{\hbar}} a_l^\dagger a_h | 0 \rangle_{\rm ev} \right] \qquad (12)$$

where $E_{\rm el}^{(0)} + E_{\rm ph}^{{\rm hf},(0)}$ can be defined as suitable energy zero in the excitation process.

Tracing up over the slow modes yields the EDOS (see Equation 3 of the main manuscript)

$$D(\omega) = \sum_{hl} {}_{\rm el}\langle 0 | a_l a_h^\dagger \delta\left(\hbar\omega - H' - V(T)\right) a_h a_l^\dagger | 0 \rangle_{\rm el} \qquad (13)$$

which depends only on the reduced Hamiltonian $H' = H - H_{\mathrm{ph}}^{\mathrm{lf}} - H_{\mathrm{el-ph}}^{\mathrm{lf}}$ (that still includes all electronic and excitonic coupling terms). The low-frequency vibrations and their EPC result in an effective disorder operator

$$V(T) = \sum_{l\lambda} \hbar\omega_\lambda g_{ll\lambda}\sqrt{(1+2\langle N_{l\lambda}\rangle)}\phi_{l\lambda} a_l^\dagger a_l + \sum_{h\lambda} \hbar\omega_\lambda g_{hh\lambda}\sqrt{(1+2\langle N_{h\lambda}\rangle)}\phi_{h\lambda} a_h a_h^\dagger. \quad (14)$$

$V(T)$ depends on the microscopic molecular parameters (EPC parameters $g_{hh\lambda}$ and $g_{ll\lambda}$) and the mode energies $\hbar\omega_\lambda$. The thermal disorder in Supplementary Equation 14 is induced by slow vibrations which contribute independently through a set of Gaussian random variables $\phi_{\dots\lambda}$ for each mode λ. $\langle N_{\dots\lambda}\rangle$ is the Bose-Einstein distribution function. We emphasise that this analytical result is exact in the indicated limit of low vibrational frequencies.

Numerically, the EDOS is obtained from calculating the spectral function with a Lanczos approach[13] and continued fraction expansion as implemented previously[14]. This is extended to the present case.

SM.2 Description of three simplified exciton models (*MUE*, *MUE+EPC*, *MUE+EC*) and the full model

It is convenient to discuss different simplified models for gaining insights. We first introduce the models and discuss the results of the EDOS simulations afterwards.

(M)anifold of (U)ncoupled (E)xcitons – This model (*MUE*) considers excitons with vanishing coupling. Vibrations are decoupled. Its Hamiltonian reads

$$H' = \sum_l \varepsilon_{ll} a_l^\dagger a_l + \sum_h \varepsilon_{hh} a_h a_h^\dagger + \sum_{hl} V_{lhhl}(\boldsymbol{R}_l - \boldsymbol{R}_h)\, a_l^\dagger a_h a_h^\dagger a_l, \quad (15)$$

where the indices $h(l)$ run over the frontier orbitals of HOMO-type (LUMO-type) centered on the respective molecular sites. This Hamiltonian is diagonal in the indices h and l since no transfer integrals are present. The non-zero matrix elements ε_{hh} (ε_{ll}) describe the ionisation energy (electron affinity) at the molecular sites located at $\boldsymbol{R}_h$ and $\boldsymbol{R}_l$. The Coulomb matrix elements $V_{lhhl}(\boldsymbol{R}_l - \boldsymbol{R}_h)$ are given by Supplementary Equation 4 implying a long-range electron-hole interaction following a Coulomb law for the spatial distances (on the fcc lattice) in C_{60}[15]. The strength of the attractive potential for the closest CT-

states is given by the material parameter V_{CT} that is obtained from *ab initio* calculations (see section SM.4). The intra-molecular excitations (for $\boldsymbol{R}_l = \boldsymbol{R}_h$) are modeled with an interaction parameter V_{ME} of the molecular exciton that amounts to 1.3 eV for C_{60} as calculated in literature[16].

MUE+EC – This model extends *MUE* by including additional electronic/excitonic coupling terms between the orbitals of holes and electrons via transfer integrals $\varepsilon_{hh'}$ [here $hh'(ll')$ describe neighboring HOMO (LUMO) indices] and coupling terms between the excitonic states via excitonic transfer integrals $t^{\text{ME}}_{lhl'h'}$ [here only transfer integrals for molecular (Frenkel) excitons are considered because of the small transition dipoles of CT excitons].

The corresponding model Hamiltonian reads

$$H' = \sum_{l,l'} \varepsilon_{ll'} a_l^\dagger a_{l'} + \sum_{h,h'} \varepsilon_{hh'} a_h a_{h'}^\dagger + \sum_{h,l} V_{lhhl}(\mathbf{R}_l - \mathbf{R}_h) a_l^\dagger a_h a_h^\dagger a_l + \sum_{h,l,h',l'} V_{lhl'h'} a_l^\dagger a_h a_{l'} a_{h'}^\dagger. \quad (16)$$

In C_{60} the transfer integrals are not very large when compared to other small-molecule systems where they can exceed 100 meV[17]. On the other hand, a large number of similar transfer integrals due to the dense packing and the spherical shape of the molecules may enable charges or excitons to delocalise over several molecular units[18,19]. We simulate the EDOS, where the electron-phonon coupling is set to zero, i.e. $V(T) = 0$, thus, no thermal disorder is present. The rotational degrees of freedom of C_{60} may still result in a variation in the transfer integrals, which we include by the orientation dependence of the electronic/excitonic transfer integrals between the molecules. Specifically, we choose randomly distributed next-neighbor transfer integrals with zero mean and Gaussian standard deviation σ_{TI} of 17 meV for all kinds of transfer integrals in the simulations, which are assigned to the next neighbors for each molecule. We find this to be a typical value for the pair coupling of C_{60} LUMO states when oriented randomly[20]. We apply this value both for donor and acceptor molecules.

MUE+EPC – This model extends *MUE* by including the vibrations and their local EPC. According to section SM.1, coupling to low-frequency modes manifests as a thermal disorder potential $V(T)$. Regarding vibrations with higher frequencies, we only consider the 0-0 transitions as discussed above. The original Hamiltonian of *MUE* is thus extended according to

$$H' \rightarrow H' + V(T), \quad (17)$$

with $V(T) = V_h(T) + V_l(T)$ where $V_l(T) = \sum_{l\lambda} \hbar\omega_\lambda g_{ll\lambda} \sqrt{(1+2\langle N_{l\lambda}\rangle)}\, \phi_{l\lambda} a_l^\dagger a_l$ describes the thermal disorder of the LUMO-type orbitals and $V_h(T) = \sum_{h\lambda} \hbar\omega_\lambda g_{hh\lambda} \sqrt{(1+2\langle N_{h\lambda}\rangle)}\, \phi_{h\lambda} a_h a_h^\dagger$ describes the thermal disorder of the HOMO-type orbitals. The quantities $\phi_{l\lambda}$ (or $\phi_{h\lambda}$ in full analogy) describe independent Gaussian random variables with standard deviation 1. The temperature dependence of the disorder is given by $\sqrt{(1+2\langle N_{l\lambda}\rangle)}$ with $\langle N_{l\lambda}\rangle$ the mean occupation of mode λ. We measure the strength of the disorder $V(T)$ by its standard deviation

$$\sigma_{\mathrm{loc}}(T) = \sqrt{\sum_\lambda (\hbar\omega_\lambda g_{ll\lambda})^2 (1+2\langle N_{l\lambda}\rangle) + (\hbar\omega_\lambda g_{hh\lambda})^2 (1+2\langle N_{h\lambda}\rangle)^2}. \quad (18)$$

This model results in an EDOS that can be written as

$$D(\omega) = \sum_{hl} {}_{\mathrm{el}}\langle 0| a_l a_h^\dagger \left(\frac{1}{\sqrt{2\pi\sigma_{\mathrm{loc}}^2}} e^{-\frac{(\hbar\omega - H')^2}{2\sigma_{\mathrm{loc}}^2}} \right) a_h a_l^\dagger |0\rangle_{\mathrm{el}}. \quad (19)$$

The EDOS in Supplementary Equation 19 represents the limit of superimposed localised excitons (see main text) whose linewidth is induced by the intra-molecular vibrations. The shape of each individual transition is a Gaussian function of width σ_{loc}.

Full model – In the full model that we employ in our simulations, all terms in the above models are included (i.e. the electronic/excitonic couplings and EPC). In this case, the Hamiltonian of *MUE+EC* is extended according to

$$H' \rightarrow H' + V(T). \quad (20)$$

The so-defined Hamiltonian represents our standard model for the simulation of the EDOS $D(\omega)$.

Simulation of EDOS and its tails for the different models – The molecular parameters for rubrene:C_{60} are calculated as described in SM.3 and SM.4. As onsite energies for the acceptor species C_{60}, we use the bulk electron affinity and ionisation potential that are known for this material $\varepsilon_{ll}^{\mathrm{A}}$ =3.50 eV and $\varepsilon_{hh}^{\mathrm{A}}$ =6.76 eV, respectively[16], while other C_{60} levels are further away by about 1 eV[21,22]. For rubrene, we calculate $\varepsilon_{hh}^{\mathrm{D}}$ =5.64 eV. The direct electron-hole interaction (at distance R_{min}) is obtained as V_{CT} =0.60 eV in bulk C_{60} medium (see SM.4 for details). For larger distances we can interpolate the distance dependence of this interaction by the Coulomb law. The vibrational parameters (including vibrational frequencies $\omega_\lambda^{\mathrm{D/A}}$

and electron-phonon couplings $g^{\mathrm{D}}_{hh\lambda}$ and $g^{\mathrm{A}}_{ll\lambda}$ are calculated in density functional theory for the respective molecules and orbitals of the rubrene:C_{60} system (see SM.3 for details).

For the rubrene:C_{60} blend we simulate the EDOS for all introduced models based on the given set of material parameters. The EDOS according to *MUE* (orange in Fig. 1b in the main text) has peak positions at the analytical energies $E^{n}_{\mathrm{CT}} = \varepsilon^{\mathrm{D}}_{hh} - \varepsilon^{\mathrm{A}}_{ll} - \frac{V_{\mathrm{CT}}}{\sqrt{n}}$ (for $n = 1, \dots, \infty$ on the fcc lattice) that is also obtained numerically at the expected energies E^{1}_{CT} =1.54 eV, E^{2}_{CT} =1.72 eV, etc.. Note that, beyond this manifold of states, the Frenkel-type excitation on C_{60} or other CT exciton states are significantly higher in energy than the lowest CT-excitons ($E^{1(2)}_{\mathrm{CT}}$) of the chosen D-A blends and are irrelevant in the present discussion of the low-energy spectrum.

We studied the different broadening mechanisms extending *MUE* by disentangling three individual contributions to the broadening of the CT exciton bands. We simulate the EDOS of the blend involving an additional but very small inhomogeneous disorder of strength $\sigma_{\mathrm{D}} = 10$ meV (green in Fig. 1b in the main text). This broadening mechanism can be understood as minimal extension of *MUE* and may describe a source of disorder, for instance, deviations from a perfect geometry.

As a second mechanism, we study the vibration-induced mechanism by low-frequency vibrations and extend *MUE* to *MUE+EPC.* The resulting EDOS (black in Fig. 1b in the main text) shows a strong broadening effect with a CT-exciton band whose tail reaches deep into the gap. This is an unavoidable consequence of the molecular motion at room temperature. The shape of the CT-exciton band for this model can be evaluated analytically and yields a Gaussian-shaped profile with standard deviation σ_{loc} (see Supplementary Equations 6 and 7 which allows us to validate the numerical results obtained with Equation 3 of the main text. For the numerical $D(\omega)$, we measure a standard deviation of $\sigma^{\mathrm{num}}_{\mathrm{loc}}$ =70.7 meV by fitting a Gaussian, which agrees with the analytical broadening σ_{loc} =68.9 meV, thus, further corroborating our numerical approach.

The third mechanism creating exciton bands is the electronic and/or excitonic coupling (*MUE+EC*) between states. Such couplings are commonly associated with the delocalisation of states and should improve charge transport and increase exciton splitting rates[18] in contrast to the case of disorder or molecular vibrations. This difference between the three mechanisms highlights the ambivalent nature of the width of CT exciton bands and requires the disentanglement of different effects. The resulting low-energy EDOS (red in Fig. 1c in the main text) exhibits a band around the energy E^{1}_{CT} which – in absence of vibrations (and hence EPC) – has an effective half band width of $B^{\mathrm{FWHM/2}}_{\mathrm{CT}} \approxeq 90$ meV. This band is

dominated by transfer integrals that are responsible for delocalisation of the electron over several LUMO-type orbitals on C_{60} molecules. In contrast, the hole wave function at the rubrene donor molecule remains almost localised. The exciton band is therefore a consequence of the high connectivity of the C_{60} molecules in the first shell around the donor leading to a rich exciton manifold despite random orientation of the molecules. Previous experiments have suggested that electrons can rapidly delocalise in a related set of CT states hosted by C_{60} clusters[18,19]. Intriguingly, although the observed band width is comparable to the case of EPC-induced bands in Fig. 1b in the main text, the tail at the low-energy side of this band is better described by an exponential energy dependence instead of a Gaussian line shape (see *MUE+EPC*). Interestingly, exponential line shapes have been reported in recent experiments on high-efficiency solar cells utilizing non-fullerene acceptors[10].

After disentangling the individual linewidth contributions, we simulated the EDOS including the intra-molecular EPC and the EC simultaneously (see blue line in Fig. 1c in the main text). We measure a total Gaussian broadening of the EDOS tail of σ_{tot} =69.7 meV that is slightly decreased compared to the value solely induced by the intra-molecular EPC. Although taking into account the 180 meV bandwidth due to the EC, we observe no increase in the CT linewidth. The decrease of the total broadening reflects the correlation of both mechanisms. However, we conclude that thermal disorder dominates the linewidth resulting in the observation of Gaussian tails for those donor-acceptor blends in agreement with experiments.

SM.3 Electron-vibration coupling for various molecular species.

Frozen phonon method for mode resolved EPC – The EPC constants and the mode energies are calculated with the *frozen phonon* method using the GAUSSIAN09 software package at the B3LYP/6-311G** level of theory[23–27] for the isolated donor molecule in the gas phase. The EPC constants $g_{hh\lambda}$ for the HOMO and $g_{ll\lambda}$ for the LUMO of C_{60} are calculated with the NWchem 6.5 software package[28] at the B3LYP/6-311G** level of theory. The mode resolved dimensionless coupling constants $g_{hh\lambda}$ (analogously for $g_{ll\lambda}$) are calculated as (see references[17,29] for details)

$$g_{hh\lambda} = \frac{1}{\sqrt{2\hbar(\omega_\lambda)^3}} \frac{\partial \varepsilon_{hh}}{\partial X_\lambda} \qquad (21)$$

where X_λ is the amplitude of the phonon normal modes in units of Å (amu)$^{1/2}$.

EPC and HOMO/LUMO degeneracy in C_{60} – The states of the isolated C_{60} molecule are degenerated by orbital symmetry with a 5-fold degeneracy of the HOMO and a 3-fold degeneracy of the LUMO and deserve some considerations. The degeneracies are slightly lifted with a frozen phonon distortion which allows us to calculate 5 different coupling constants $g_{hh\lambda}$ and 3 different coupling constants $g_{ll\lambda}$ for each individual mode λ. The EPC of a single C_{60} molecule is therefore given as

$$\hat{H}_{\text{el-ph}}^{C_{60}} = \sum_\lambda \hbar\omega_\lambda \left(b_\lambda^\dagger + b_\lambda\right)\left(\sum_{l=1}^{3} g_{ll\lambda} a_l^\dagger a_l + \sum_{h=1}^{5} g_{hh\lambda} a_h a_h^\dagger\right) \quad (22)$$

To describe the vibration-induced bandwidth of donor-acceptor CT-states and of excitonic states in pristine C_{60}, an efficient description (based on effective single HOMO and LUMO states in consistency to the general electronic model) can be worked out with an averaged coupling. For an electronic state equally weighting the 3 LUMO orbitals according to $\left|\psi_{C_{60}}^{\text{LUMO}}\right\rangle = \frac{1}{\sqrt{3}}\sum_{l=1}^{3} a_l^\dagger \left|\psi^{(0)}\right\rangle$, we obtain

$$\hat{H}_{\text{el-ph}}^{C_{60}} \rightarrow \sum_\lambda \hbar\omega_\lambda g_{ll\lambda}^{\text{eff}} \left(b_\lambda^\dagger + b_\lambda\right) a_l^\dagger a_l \quad (23)$$

with $g_{ll\lambda}^{\text{eff}} = \sum_{k=1}^{3} \frac{g_{kk\lambda}}{3}$ an effective coupling from an average of the three LUMO couplings of a given molecule. The analogous procedure applies for the HOMOs. Consequently, for a molecular exciton on a single C_{60} molecule the vibronic coupling results from an equal-weighted superposition of all 15 HOMO-LUMO combinations $\left|\psi_{C_{60}}^{\text{ME}}\right\rangle = \left|\psi_{C_{60}}^{\text{LUMO}}\right\rangle\left|\psi_{C_{60}}^{\text{HOMO}}\right\rangle$ of the product states.

We emphasise that the EPC induced broadening of the individual orbitals is different from the broadening of the molecular exciton. The individual HOMO and LUMO contributions lead to a total broadening of

$$\left(\sigma_{C_{60}}^{\text{LUMO}}\right)^2 + \left(\sigma_{C_{60}}^{\text{HOMO}}\right)^2 = \sum_\lambda \left(\hbar\omega_\lambda g_{ll\lambda}^{\text{eff}}\right)^2 (1 + 2\langle N_{l\lambda}\rangle) + \left(\hbar\omega_\lambda g_{hh\lambda}^{\text{eff}}\right)^2 (1 + 2\langle N_{h\lambda}\rangle)^2 \quad (24)$$

which is equivalent to Supplementary Equation 18. In particular for CT-states we obtain an EPC induced broadening according to Supplementary Equation 24 for both donor-C_{60} and C_{60}-C_{60} CT-states using the effective EPC constants $g_{ll\lambda}^{\text{eff}}$ and $g_{hh\lambda}^{\text{eff}}$ for the C_{60} orbitals. In contrast, for the molecular exciton in C_{60} we find

$$\left(\sigma_{C_{60}}^{\text{ME}}\right)^2 = \sum_\lambda (\hbar\omega_\lambda)^2 (1 + 2\langle N_\lambda\rangle)\left(g_{ll\lambda}^{\text{eff}} + g_{hh\lambda}^{\text{eff}}\right)^2. \quad (25)$$

This differs from Supplementary Equation 24 and might lead to an enhanced or reduced broadening depending on the relative sign of the coupling constants $g_{ll\lambda}^{\text{eff}}$ and $g_{hh\lambda}^{\text{eff}}$ i.e. the broadening might be partly cancelled if $g_{ll\lambda}^{\text{eff}} = -g_{hh\lambda}^{\text{eff}}$ for a certain mode λ.

SM.4 *Ab initio* calculations of polarisation and CT-energies for donor:C_{60} blends

Equations for the CT-energy, exciton binding energy, and polarisation corrections – The aim of this section is to obtain the electron-hole interaction energy V_{CT} and the charge-transfer energy E_{CT} of a donor-acceptor pair in the organic film and to describe the approach to their simulation. We consider that this pair is diluted in the host material, which is taken to be C_{60} fullerenes. In general, the theoretical CT-energy $E_{\mathrm{CT}}^{\mathrm{theo}}$ is related to V_{CT} as

$$E_{\mathrm{CT}}^{\mathrm{theo}} = IP_{\mathrm{s}}(\mathrm{D}) - EA_{\mathrm{s}}(\mathrm{A}) - V_{\mathrm{CT}}. \tag{26}$$

Here $IP_{\mathrm{s}}(\mathrm{D})$ is the ionisation potential of the donor (D) and $EA_{\mathrm{s}}(\mathrm{A})$ the electron affinity of the acceptor (A). Both are taken in an environment that is mainly composed of acceptor molecules (the surrounding is indicated by the subscript s that can either refer to the donor (D) or the acceptor (A) material). These quantities are understood as the peak center positions in contrast to onset energies that are frequently used in corresponding photoemission measurements (see below). The difference $IP_{\mathrm{s}}(D) - EA_{\mathrm{s}}(A)$ is sometimes called the transport gap, which is to be distinguished from the excitonic gap[30]. The energy of the latter gets close to the energy of the former when separating electron and hole in the exciton. V_{CT} describes the screened Coulomb interaction between hole and electron at the donor-acceptor pair distance (see Supplementary Equation 4). The individual contributions in Supplementary Equation 26 are extracted from density-functional theory (DFT) simulations.

We focus first on the simulation of V_{CT} for a relaxed geometry of a donor-acceptor dimer. The simulations are performed in gas phase and polarisation corrections are subsequently added to account for the environmental screening

$$V_{\mathrm{CT}} = \mathit{\Delta}_0 - P_{\mathrm{CT}}. \tag{27}$$

Here Δ_0 is the electron-hole interaction energy in the gas phase, which is calculated for a fixed dimer geometry as

$$\begin{aligned}\mathit{\Delta}_0 &= IP_{\mathrm{D}^+}(\mathrm{A}^-) - EA_{\mathrm{D}^0}(\mathrm{A}^0)\\ &= \big(E(\mathrm{D}^+,\mathrm{A}^0) - E(\mathrm{D}^+,\mathrm{A}^-)\big) - \big(E(\mathrm{D}^0,\mathrm{A}^0) - E(\mathrm{D}^0,\mathrm{A}^-)\big)\end{aligned} \tag{28}$$

We transform this equation slightly to

$$\mathit{\Delta}_0 = IP_{\mathrm{A}^0}(\mathrm{D}^0) - E_{\mathrm{CT}}^{\mathrm{vac}} - EA_{\mathrm{D}^0}(\mathrm{A}^0) \tag{29}$$

with the CT energy for the dimer in vacuum

$$E_{\mathrm{CT}}^{\mathrm{vac}} = E(\mathrm{D}^{+}, \mathrm{A}^{-}) - E(\mathrm{D}^{0}, \mathrm{A}^{0}). \quad (30)$$

The involved energies $E(\dots, \dots)$ in Supplementary Equations 28 to 30 represent the DFT total energies of the dimer for a certain charging state of the donor (D) and the acceptor C_{60} (A) as indicated by superscripts to the species. The correct charging is ensured by using constrained DFT[31] within the NWchem 6.5 package. The energies were calculated with the CAM-B3LYP/6-311G** level of theory[32,33] and the additional inclusion of Grimme's empirical dispersion correction GD3[34] and asymptotic correction LB94[35].

To obtain V_{CT}, we simulate the polarisation correction to Δ_0 namely P_{CT}. According to the three energy terms in Supplementary Equation 29, P_{CT} has three contributions:

$$P_{\mathrm{CT}} = P_{\mathrm{A}}^{\mathrm{dimer}}(\mathrm{D}) - P_{\mathrm{A}}^{\mathrm{dimer}}(\mathrm{DA}) + P_{\mathrm{A}}^{\mathrm{dimer}}(\mathrm{A}), \quad (31)$$

where the parentheses indicate the molecules whose charging state is changed. For instance, the energy $IP_{\mathrm{A}^0}(\mathrm{D}^0)$ in Supplementary Equation 29 is corrected by the polarisation energy $-P_{\mathrm{A}}^{\mathrm{dimer}}(\mathrm{D})$. The superscript recalls that the polarisation contributions are due to the environment of the dimer. The actual calculations are described below.

Finally, we relate the first two terms in Supplementary Equation 26 to their gas-phase values by introducing the respective polarisation corrections

$$IP_{\mathrm{A}}(\mathrm{D}) = IP_{\mathrm{gas}}(\mathrm{D}) - P_{\mathrm{A}}(\mathrm{D}) \quad (32)$$

$$EA_{\mathrm{A}}(\mathrm{A}) = EA_{\mathrm{gas}}(\mathrm{A}) - P_{\mathrm{A}}(\mathrm{A}). \quad (33)$$

Combining Supplementary Equations 26, 27, 31 to 33, we find the CT-energy as

$$E_{\mathrm{CT}}^{\mathrm{theo}} = IP_{\mathrm{gas}}(\mathrm{D}) - \left(P_{\mathrm{A}}(\mathrm{D}) - P_{\mathrm{A}}^{\mathrm{dimer}}(\mathrm{D})\right)$$
$$- EA_{\mathrm{gas}}(\mathrm{A}) - \left(P_{\mathrm{A}}(\mathrm{A}) - P_{\mathrm{A}}^{\mathrm{dimer}}(\mathrm{A})\right) - \Delta_0 - P_{\mathrm{A}}^{\mathrm{dimer}}(\mathrm{DA}) \quad (34)$$

In subsequent sections, we discuss the approaches to the individual terms in Supplementary Equation 34.

Gas-phase energies – The gas-phase EA of C_{60} of $EA_{\mathrm{gas}}(\mathrm{A})$ =2.68 eV and $P_{\mathrm{A}}(\mathrm{A})$ =0.82 eV are known from previous works[16,36] yielding the C_{60} bulk value of $EA_{\mathrm{A}}(\mathrm{A})$ =3.5 eV. The gas-phase IP of rubrene is known from experiment[8]. The gas-phase IPs of the other donor molecules TAPC, Spiro-MeO-TPD and m-MTDATA are unknown and need to be calculated from the respective experimental thin film values by

considering the bulk polarisation in such films. That is we calculate the gas-phase ionisation potential $IP_{\text{gas}}(\text{D}) = IP_{\text{D}}(\text{D}) + P_{\text{D}}(\text{D})$ from the ionisation potential $IP_{\text{D}}(\text{D})$ in the film and add its associated polarisation correction $P_{\text{D}}(\text{D})$. The $IP_{\text{D}}(\text{D})$ are taken from photoemission data, hence we need to include the surface effect in the polarisation correction $P_{\text{D}}(\text{D})$ since the signal is dominated by surface donor molecules (small inelastic mean free path of electrons).

To model the surface sensitivity of UPS, the excess charges in the charged configuration contributing to $IP_{\text{D}}(\text{D})$ are assumed to originate from donor molecules in the surface layer and one sub-surface layer with equal probability. To calculate the surface effect, we adopt a cylinder geometry where the excess charge of a molecule is inside the cylinder (of polar radius R_{cyl} and a height of $2R_{\text{cyl}}$) that has the same volume V as the molecule. The molecular volume V is calculated based on the geometrical extend of the molecule according to its principal semi-axes a, b, c which takes into account a non-spherical shape. The corresponding volume of an ellipsoid $V = \frac{4}{3}\pi abc$ that contains the molecule is equated to the volume of the cylinder of $V = 2\pi R_{\text{cyl}}^3$. Hence, the radius R_{cyl} is calculated as $R_{\text{cyl}} = \left(\frac{2}{3}abc\right)^{\frac{1}{3}}$. This leads to a monopole polarisation correction of $P_{\text{mono}}^{\text{surface}} = \left(1 - \frac{1}{\varepsilon}\right)\frac{E_{\text{H}}}{2}\frac{a_0}{R_{\text{cyl}}}\left(\frac{1}{3} + \frac{\pi}{8}\right)$ caused by the molecules below the surface. The dielectric constant ε for m-MTDATA is extracted from experiment[7], while for the other two molecules we take a value of $\varepsilon = 3$ that is typical for organic molecules[5,6]. In Supplementary Table 1, we summarise the results obtained for the donor gas-phase $IP_{\text{gas}}(\text{D})$.

Bulk polarisation corrections for monomers – We calculate the polarisation correction to the charging energies of single molecules $P_{\text{A}}(\text{D})$ in a bulk acceptor environment. The monopole polarisation correction for the respective excess charge is computed in a continuum approach[16] as

$$P_{\text{mono}} = \left(1 - \frac{1}{\varepsilon}\right)\frac{E_{\text{H}}}{2}\frac{a_0}{R} \tag{35}$$

where ε is the dielectric constant (relative permittivity) of the continuum either composed of donor or acceptor molecules. We assume the continuum being outside a sphere with radius R that contains the same volume V as the respective molecule. The volume V is calculated based on the geometrical extend of the molecule according to its principal semi-axes a, b, c. Thus R is calculated as the geometric mean of the principal semi-axis a, b, c as $R = (abc)^{\frac{1}{3}}$.

Bulk polarisation corrections for dimers – The dipole polarisation correction $P_{\mathrm{A}}^{\mathrm{dimer}}(\mathrm{DA})$ in Supplementary Equation 31 of the excited dimer composed of a negatively charged C_{60} and a positively charged donor in the C_{60} environment is calculated within the continuum approach[16] as

$$P_{\mathrm{dip}} = \left(1 - \frac{1}{\varepsilon}\right) \frac{E_{\mathrm{H}}}{3} \frac{p^2 a_0}{R^3}. \quad (36)$$

ε is the dielectric constant of C_{60} which is set to 4.4[37]. p is the electric dipole moment of the dimer, which is calculated from the respective Löwdin-charges during the NWchem single-point calculation. Finally, R is the radius of a sphere that contains the same volume V as the dimer. The dimer volume is calculated based on its principal semi-axes a, b, c as the volume of the corresponding ellipsoid.

The polarisation correction of the monopole charges in the dimer geometry is given by the quantities $P_{\mathrm{A}}^{\mathrm{dimer}}(\mathrm{D})$ and $P_{\mathrm{A}}^{\mathrm{dimer}}(\mathrm{A})$, where we use Supplementary Equation 35 with the radius obtained from the dimer volume.

Donor geometries for rubrene, TAPC, m-MTDATA and Spiro-MeO-TPD – The donor-C_{60} geometries for the rather small molecules rubrene and TAPC were obtained by DFT-relaxations using the GAUSSIAN16 package and the CAM-B3LYP/3-21G level of theory. To account for the different configurations that are possible, we relaxed three different initial configurations and chose the geometry with the smallest final energy as the representative configuration for which $E_{\mathrm{CT}}^{\mathrm{theo}}$ was calculated.

For the molecules that we select as examples for larger and more flexible ones, namely Spiro-MeO-TPD and m-MTDATA, a larger configurational space is relevant[9]. The representative set of dimer configurations for these donor-C_{60} systems is therefore extracted from molecular dynamics (MD) simulations. Clusters of single donor molecules surrounded by minimum five C_{60} molecules are constructed and simulated at T =300 K using density-functional based tight-binding (DFTB) as implemented in the DFTB+ software package[38–44]. After an equilibration run for 10 ps in the NVT ensemble using a Nosé-Hoover chain thermostat with a coupling strength of 30 cm^{-1} (3.7 meV)[45–47], a production run is performed for 30 ps in the NVE ensemble.

Distribution of CT-energies for highly flexible molecules – Based on the obtained MD trajectories for m-MTDATA and Spiro-MeO-TPD, we evaluate the properties of the CT-states with C_{60} as acceptor. We calculate the excitation energies and oscillator strengths of the first three excited states for all donor-C_{60}

dimers in the cluster by performing time-dependent density functional theory (TD-DFT) calculations along the trajectory (each 50 fs). The GAUSSIAN09 software package was used at the CAM-B3LYP/3-21G level of theory[23]. Based on this dense sampling of configurations, the distribution of excitation lines was obtained and yields a Gaussian shape that is centered at $E_{\mathrm{CT,peak}}^{\mathrm{theo,MD}}$ with a standard deviation $\sigma_{\mathrm{tot}}^{\mathrm{MD}}(T)$ of 170 meV for m-MTDATA and 126 meV for Spiro-MeO-TPD. We observe that the width of this distribution for both highly flexible molecules is larger than the width $\sigma_{\mathrm{loc}}(T)$ in Supplementary Equation 18 obtained from the EPCs of the molecular vibrations (cf. section SM.3), thus confirming our expectation.

The larger width is expected for two reasons. Firstly, in the MD simulations, all normal modes are treated classically through the semiclassical motion of the nuclei which is comparable to the high-temperature limit. In this limit, they contribute with a broadening $\sigma_{\mathrm{loc}}^{\mathrm{class}}(T) = \sqrt{2\Lambda_{\mathrm{DA}} k_{\mathrm{B}} T}$ with $\Lambda_{\mathrm{DA}} = \Lambda_{\mathrm{D}} + \Lambda_{\mathrm{A}} = \sum_\lambda \hbar\omega_\lambda (g_{hh\lambda})^2 + \sum_\lambda \hbar\omega_\lambda (g_{ll\lambda})^2$ the full relaxation energy of the donor-acceptor complex according to Marcus theory[9]. Secondly, the MD simulations also include effects beyond the standard EPC modelling (such as anharmonic effects of vibrational modes or further conformational effects). To obtain the strength of these effects, we calculate the difference between the total broadening $\sigma_{\mathrm{tot}}^{\mathrm{MD}}(T)$ at 300 K and the conventional EPC-induced width $\sigma_{\mathrm{loc}}^{\mathrm{class}}(T)$. We find that these effects amount to 26% of the EPC-induced width $\sigma_{\mathrm{loc}}^{\mathrm{class}}(T)$ for m-MTDATA (24% for Spiro-MeO-TPD) and we can describe the total broadening according to $\sigma_{\mathrm{tot}}(T) = \alpha\sigma_{\mathrm{loc}}(T)$ with factor α=1.26 for m-MTDATA and α=1.24 for Spiro-Meo-TPD.

We finally discuss the center of the CT energy distribution that is obtained by the above combined MD and TD-DFT approach. Due to the excited state properties being calculated for the individual donor-acceptor dimers in gas phase, it lacks the polarization by the film medium which leads to an energy offset Δ_{MD} as compared to the energies obtained from the approach in Supplementary Equation 34. Additional contributions to Δ_{MD} may arise from systematic errors of the TD-DFT approach including basis set effects as well as molecular deformations. We therefore obtain the offset by a difference calculation based on a representative geometry and shift the distribution of excitation energies according to $E_{\mathrm{CT}}^{\mathrm{theo}} = E_{\mathrm{CT,peak}}^{\mathrm{theo,MD}} + \mathit{\Delta}_{\mathrm{MD}}$. We chose the donor-$C_{60}$ dimer with the lowest excitation energy and highest oscillator strength as the representative dimer geometry and calculate $\mathit{\Delta}_{\mathrm{MD}}$ from the difference in the CT-energies between both methods (Supplementary Equation 34 and TD-DFT approach). The resulting peak energy is taken as the CT energy for the simulation of the EDOS $D(\omega)$, while the width of the distribution is unaffected by this shift.

In Supplementary Table 2 we summarise the results for all relevant material parameters based on this combined approach and compare the CT-peak positions to the experimental values. For the donor species m-MTDATA and Spiro-MeO-TPD we calculate V_{CT} from $V_{\mathrm{CT}} = IP_{\mathrm{A}}(\mathrm{D}) - EA_{\mathrm{A}}(\mathrm{A}) - E_{\mathrm{CT}}^{\mathrm{theo}}$.

In Supplementary Table 3 we summarise the obtained material parameters taken for the simulation of the EDOS $D(\omega)$.

SM.5 Analytical model for maximum open-circuit voltage V_{r}

Open-circuit voltage – The open-circuit voltage for a solar cell is given by[48,49]

$$V_{\mathrm{OC}} = \frac{k_{\mathrm{B}}T}{q} \ln\left(\frac{J_{\mathrm{SC}}}{J_0} + 1\right). \tag{37}$$

Here J_{SC} is the charge current at short-circuit conditions and J_0 is the dark saturation current. An upper limit for the open-circuit voltage is[50]

$$V_{\mathrm{r}} = \frac{k_{\mathrm{B}}T}{q} \ln\left(\frac{J_{\mathrm{SC}}}{J_{\mathrm{em,0}}} + 1\right) \tag{38}$$

which only accounts for radiative contributions to the open-circuit voltage and neglects non-radiative effects. Here $J_{\mathrm{em,0}}$ represents a current leading to the emission of thermal photons. The charge currents J_{SC} and $J_{\mathrm{em,0}}$ are related to the photovoltaic quantum efficiency $\Sigma(E)$ via

$$J_{\mathrm{SC}} = q \int_0^{\infty} \mathrm{d}E \; \phi_{\mathrm{Sun}}(E)\, \Sigma(E) \tag{39}$$

$$J_{\mathrm{em,0}} = q \int_0^{\infty} \mathrm{d}E \; \phi_{\mathrm{BB}}^{T}(E)\, \Sigma(E) \tag{40}$$

where $\phi_{\mathrm{Sun}}(E)$ and $\phi_{\mathrm{BB}}^{T}(E)$ are the respective photon fluxes of the sun and the ambient at temperature T. $\Sigma(E)$ is accessible from our simulations of the EDOS $D(\omega)$ which allows the numerical calculation of V_{r} (see Equation 4 and Fig. 3a in the main text) and the comparison with the experiment[51] (see Fig. 3b in the main text). Here, we derive a simple analytical model to investigate how the absorption tail affects V_{r}.

Shockley-Queisser-model for $\overline{V}$ and extension to a broadened absorption tail – To quantify the radiative losses of the open-circuit voltage V_{r} due to a finite absorption tail, we study the following analytical model. Firstly, we calculate numerically the radiative limit of the open-circuit voltage in the Shockley-Queisser

(SQ) model with a gap energy E_g defined as the absorption maximum of the lowest CT-peak, i.e. $E_\mathrm{g} = E_\mathrm{CT,peak} = E_\mathrm{CT}^1$. In this limit the photovoltaic external quantum efficiency $\Sigma(E)$ is given by a step function with an infinitely steep absorption edge at E_g

$$\Sigma(E) = \begin{cases} 1 & E \geq E_\mathrm{g} \\ 0 & E < E_\mathrm{g} \end{cases} \tag{41}$$

In the radiative limit, the open-circuit voltage of the SQ-model becomes

$$V_\mathrm{r}^\mathrm{SQ} = \frac{k_\mathrm{B}T}{q}\ln\left(\frac{J_\mathrm{SC}^\mathrm{SQ}}{J_0^\mathrm{SQ}} + 1\right) \tag{42}$$

with the short-circuit current from the absorbed solar photons

$$J_\mathrm{SC}^\mathrm{SQ} = q\int_{E_\mathrm{g}}^{\infty} \mathrm{d}E\ \phi_\mathrm{Sun}(E) \tag{43}$$

and the dark saturation current due to the emitted thermal photons according to

$$J_\mathrm{em,0}^\mathrm{SQ} = q\int_{E_\mathrm{g}}^{\infty} \mathrm{d}E\ \phi_\mathrm{BB}^T(E). \tag{44}$$

Secondly, in presence of a broadening mechanism (e.g. induced by the EPC of intra-molecular modes) that smears out the absorption edge, the open-circuit voltage is reduced below the limiting V_r^SQ. To study this effect for our results for the temperature dependent broadening $\sigma(T)$ of the EDOS, we extend the SQ-model for the EQE by adding a tail according to

$$\Sigma(E) = \begin{cases} 1 & E \geq E_\mathrm{g} \\ e^{-\frac{(E-E_\mathrm{g})^2}{2\sigma^2(T)}} & E < E_\mathrm{g} \end{cases} \tag{45}$$

The short circuit and the dark saturation currents then become

$$J_\mathrm{SC} = J_\mathrm{SC}^\mathrm{tail} + J_\mathrm{SC}^\mathrm{SQ} \tag{46}$$

$$J_\mathrm{em,0} = J_\mathrm{em,0}^\mathrm{tail} + J_\mathrm{em,0}^\mathrm{SQ} \tag{47}$$

with

$$J_\mathrm{SC}^\mathrm{tail} = \int_0^{E_\mathrm{g}} \mathrm{d}E\ \phi_\mathrm{Sun}(E)e^{-\frac{(E-E_\mathrm{g})^2}{2\sigma^2(T)}} \tag{48}$$

$$J_\mathrm{em,0}^\mathrm{tail} = \int_0^{E_\mathrm{g}} \mathrm{d}E\ \phi_\mathrm{BB}^T(E)e^{-\frac{(E-E_\mathrm{g})^2}{2\sigma^2(T)}}. \tag{49}$$

Finally, the radiative open-circuit voltage takes the form

$$V_{\mathrm{r}}^{\mathrm{SQ+t}} = V_{\mathrm{r}}^{\mathrm{SQ}} - \Delta V_{\mathrm{r}} \tag{50}$$

where the deviation from the SQ-limit can be expressed as

$$\Delta V_{\mathrm{r}} = \frac{k_{\mathrm{B}}T}{q}\ln\left(1+\frac{J_{\mathrm{em,0}}^{\mathrm{tail}}}{J_{\mathrm{em,0}}^{\mathrm{SQ}}}\right) - \frac{k_{\mathrm{B}}T}{q}\ln\left(1+\frac{J_{\mathrm{SC}}^{\mathrm{tail}}-J_{\mathrm{em,0}}^{\mathrm{SQ}}}{J_{\mathrm{SC}}^{\mathrm{SQ}}+J_{\mathrm{em,0}}^{\mathrm{SQ}}}\right). \tag{51}$$

Here, a voltage gain that arises due to the presence of a finite absorption tail (second term on the rhs. of Supplementary Equation 51) is counteracted by radiative voltage losses in the first term on the rhs. of Supplementary Equation 51. Supplementary Equations 50 and 51 are used for the numerical calculations in Fig. 3c, 3d, 4c and Fig. 5a of the main text.

Analytical expression for $\overline{V}$and quantification of the losses $\boldsymbol{\Delta V_{\mathrm{r}}}$ – In Supplementary Equation 51 the voltage gain arises due to the additional photo-current $J_{\mathrm{SC}}^{\mathrm{tail}}$. However, in solar cells this contribution should be considered small i.e. $\frac{J_{\mathrm{SC}}^{\mathrm{tail}}-J_{\mathrm{em,0}}^{\mathrm{SQ}}}{J_{\mathrm{SC}}^{\mathrm{SQ}}+J_{\mathrm{em,0}}^{\mathrm{SQ}}} \approxeq \frac{J_{\mathrm{SC}}^{\mathrm{tail}}}{J_{\mathrm{SC}}^{\mathrm{SQ}}} \ll 1$. In contrast, the voltage losses due to the increased dark saturation current may reduce substantially the radiative open-circuit voltage. We expect $\frac{J_{\mathrm{em,0}}^{\mathrm{tail}}}{J_{\mathrm{em,0}}^{\mathrm{SQ}}} \gg 1$ and thus $\Delta V_{\mathrm{r}} = \frac{k_{\mathrm{B}}T}{q}\ln\left(\frac{J_{\mathrm{em,0}}^{\mathrm{tail}}}{J_{\mathrm{em,0}}^{\mathrm{SQ}}}\right)$. The open-circuit voltage can then be approximated as

$$V_{\mathrm{r}}^{\mathrm{SQ+t}} = V_{\mathrm{r}}^{\mathrm{SQ}} - \frac{k_{\mathrm{B}}T}{q}\ln\left(\frac{J_{\mathrm{em,0}}^{\mathrm{tail}}}{J_{\mathrm{em,0}}^{\mathrm{SQ}}}\right). \tag{52}$$

The above equations can be simplified since the dark saturation currents of the absorption tail $J_{\mathrm{em,0}}^{\mathrm{tail}}$ and of the SQ-model $J_{\mathrm{em,0}}^{\mathrm{SQ}}$ can be treated analytically. Firstly, we substitute the thermal black-body photon spectrum with $\phi_{\mathrm{BB}}^{T}(E) = \frac{4\pi}{c^2h^3}\frac{E^2}{e^{\beta E}-1} \rightarrow \frac{4\pi}{c^2h^3}E^2e^{-\beta E}$ since βE ≫1 for energies above the gap energy $E > E_{\mathrm{g}}$.

We therefore find for $J_{\mathrm{em,0}}^{\mathrm{SQ}}$

$$\int_{E_{\mathrm{g}}}^{\infty}\mathrm{d}E\ \frac{4\pi}{c^2h^3}E^2e^{-\beta E} = \frac{4\pi E_{\mathrm{g}}^3}{c^2h^3}e^{-\beta E_{\mathrm{g}}}\left(\frac{1}{\beta E_{\mathrm{g}}}+\frac{2}{(\beta E_{\mathrm{g}})^2}+\frac{2}{(\beta E_{\mathrm{g}})^3}\right), \tag{53}$$

which is dominated by the leading term

$$J_{\mathrm{em,0}}^{\mathrm{SQ}} \approx \frac{4\pi}{c^2h^3}E_{\mathrm{g}}^2k_{\mathrm{B}}Te^{-\beta E_{\mathrm{g}}}. \tag{54}$$

For the dark saturation current of the Gaussian tail we have

$$J_{\mathrm{em},0}^{\mathrm{tail}} = \frac{4\pi}{c^2h^3} e^{\frac{\beta^2\sigma^2(T)}{2} - \beta E_\mathrm{g}} \int_0^{E_\mathrm{g}} \mathrm{d}E\, E^2\, e^{-\frac{\left(E - E_\mathrm{g} + \sigma^2(T)\beta\right)^2}{2\sigma^2(T)}} \quad (55)$$

where the energy integral is solved explicitly with the result

$$J_{\mathrm{em},0}^{\mathrm{tail}} = \frac{4\pi}{c^2h^3} e^{\frac{\beta^2\sigma^2(T)}{2} - \beta E_\mathrm{g}} \sigma^3(T) f\left(E_\mathrm{g}, \sigma(T), \beta\right). \quad (56)$$

where

$$f\left(E_\mathrm{g}, \sigma, \beta\right) = e^{-\frac{\sigma^2\beta^2}{2}}\left(2\frac{E_\mathrm{g}}{\sigma} - 3\sigma\beta\right) + 3\left(\sigma\beta - \frac{E_\mathrm{g}}{\sigma}\right) e^{-\frac{\left(\frac{E_\mathrm{g}}{\sigma} - \sigma\beta\right)^2}{2}}$$
$$+ \sqrt{\frac{\pi}{2}}\left(1 + \left(\frac{E_\mathrm{g}}{\sigma} - \sigma\beta\right)^2\right)\left(\mathrm{erf}\left(\frac{\sigma\beta}{\sqrt{2}}\right) + \mathrm{erf}\left(\frac{\frac{E_\mathrm{g}}{\sigma} - \sigma\beta}{\sqrt{2}}\right)\right).$$

(44)(57)

The voltage loss ΔV_r depends on the linewidth $\sigma^2(T)$ as follows

$$\Delta V_\mathrm{r} = \frac{1}{q}\left(\frac{\sigma^2(T)}{2k_\mathrm{B}T} + k_\mathrm{B}T \ln\left(\frac{\sigma^3(T)}{E_\mathrm{g}^2 k_\mathrm{B}T}\right) + k_\mathrm{B}T \ln\left(f\left(E_\mathrm{g}, \sigma(T), \beta\right)\right)\right).$$

(45)(58)

We now consider the limit of small and large broadening $\sigma(T)$ leading to qualitatively different results for the voltage losses ΔV_r. For large broadening i.e. $k_\mathrm{B}T \ll \sigma(T) \leq E_\mathrm{g}$ we find

$$\Delta V_\mathrm{r} = \frac{k_\mathrm{B}T}{q} \ln\left(\frac{\sigma^2(T)}{E_\mathrm{g} k_\mathrm{B}T}\right) \quad (59)$$

whereas for small broadening i.e. $k_\mathrm{B}T \leq \sigma(T) \ll E_\mathrm{g}$ or $\sigma(T) \leq k_\mathrm{B}T \ll E_\mathrm{g}$, we find

$$\Delta V_\mathrm{r} = \frac{1}{q}\left(\frac{\sigma^2(T)}{2k_\mathrm{B}T} + k_\mathrm{B}T \ln\left(\sqrt{\frac{\pi}{2}} \frac{\sigma(T)}{k_\mathrm{B}T}\right)\right). \quad (60)$$

SM.6 Systems with non-fullerene acceptors

EDOS simulations in non-fullerene systems – In the next paragraphs we discuss the material parameters and the subsequent EDOS simulations involving the non-fullerene acceptor materials boron subnaphtalocyanine chloride (SubNc), boron subphthalocyanine chloride (SubPc), and chlorinated SubPc (Cl_6-SubPc).

Energy levels in SubNc, SubPc and Cl_6-SubPc – For the non-fullerene acceptor materials SubNc, SubPc and Cl_6-SubPc we use reference values for the ionisation potential and the electron affinity from the respective peak positions of UPS and IPES measurements[52] and correct these values by a surface polarisation of 0.15 eV each, thus leading to the polarisation corrected bulk values of ε_{hh}=5.7 eV and ε_{ll}=2.65 eV for SubNc and ε_{hh}=6.15 eV and ε_{ll}=2.9 eV for SubPc. For Cl_6-SubPc we estimate the orbital energies based on those of SubPc and account for a chlorination shift of 300 meV[53]. This yields a fundamental electronic gap of $\varepsilon_{hh} - \varepsilon_{ll} =$ 3.05 eV (3.25 meV for SubPc and Cl_6-SubPc) for free electron-hole pairs that already includes the reorganisation energy from the high-frequency vibrational modes. From the comparison to the absorption peak at 1.75 eV of SubNc we determine the binding energy of the molecular exciton to be V_{ME} =1.3 eV. The experimental absorption maximum of neat SubPc lies at 2.08 eV (see Fig. 5b in the main text) yielding a binding energy of V_{ME} =1.17 eV. The absorption maximum of Cl_6-SubPc is slightly lower due to chlorination as compared to SubPc[54] at 2.05 eV (see Table S24 of the supporting material in the according reference). The binding energy of the molecular exciton in Cl_6-SubPc then is given by V_{ME} =1.2 eV.

Material parameters for the EPC and EC in SubNc, SubPc and Cl_6-SubPc – We calculated the EPC in SubNc, SubPc and Cl_6-SubPc using the method described in section SM.3.

Furthermore we calculate the relevant transfer integrals according to the respective crystal structure of SubNc[54], SubPc[55], and Cl_6-SubPc[56]. The transfer integrals for electrons $\varepsilon_{ll'}$ (and holes in full analogy) are calculated in DFT based on all relevant dimers according to the method presented in literature[57]. They are obtained as the matrix elements of the Hamiltonian with respect to the LUMO states $|\psi_l\rangle$ of the SubNc monomers as

$$\varepsilon_{ll'} = \langle\psi_l|H|\psi_{l'}\rangle. \tag{61}$$

Note that the monomer orbitals $|\Phi_l\rangle$ calculated in the DFT-simulations are in general not orthogonal. Hence a Löwdin orthogonalisation $|\psi_l\rangle = S^{-\frac{1}{2}}|\Phi_l\rangle$ is performed to orthogonalise them

$$\varepsilon_{ll'} = \langle\Phi_{l'}|S^{-\frac{1}{2}}FS^{-\frac{1}{2}}|\Phi_l\rangle, \tag{62}$$

where F is the Hamiltonian of the dimer (Fock matrix) and S the overlap matrix of the non-orthogonal monomer orbitals $S_{ll'} = \langle\Phi_l|\Phi_{l'}\rangle$. The Hamiltonian F, the overlap matrix S and the monomer orbitals

$|\Phi_l\rangle$ were obtained by DFT-calculations with the Gaussian16 package[58] and the B3LYP level of theory combined with the 6-311G** basis set.

Compensation of the EPC induced HOMO/LUMO broadening in SubNc, SubPc and Cl_6-SubPc – For SubNc we calculate an EPC induced Gaussian broadening of $\sigma_{\mathrm{SubNc}}^{\mathrm{HOMO}} = 38.8$ meV for the HOMO and $\sigma_{\mathrm{SubNc}}^{\mathrm{LUMO}} = 48.9$ meV for the LUMO. The combination of both orbitals according to Supplementary Equation 24 would result in a total linewidth of $\sqrt{\left(\sigma_{\mathrm{SubNc}}^{\mathrm{HOMO}}\right)^2 + \left(\sigma_{\mathrm{SubNc}}^{\mathrm{LUMO}}\right)^2} = 62.4$ meV. However, for the molecular exciton we obtain $\sigma_{\mathrm{SubNc}}^{\mathrm{ME}} = 44.4$ meV according to Supplementary Equation 25, i.e. the broadening of the molecular excitons is reduced by $\Delta\sigma_{\mathrm{SubNc}} = 18.0$ meV (-29% of the broadening of the individual contributions) leading to reduced voltage losses induced by the smaller linewidth. The reason for this observation is the compensation of the EPC to both frontier levels due to the similar shape of the orbitals on a single SubNc molecule. This behaviour is reflected in Supplementary Equation 25 where the broadening may vanish if the molecular EPC constants have same values and opposite sign.

Moreover, we find that SubNc is not an exception for this compensation effect. In Supplementary Table 5 we briefly summarise the calculated values for the EPC-induced broadening of the individual molecular orbitals with that of molecular excitons for SubNc, SubPc and Cl_6-SubPc. All obtained values support our design strategy that the EPC-induced broadening may be significantly reduced for molecular excitons as compared to the individual contributions for electrons and holes (which would enter the broadening of e.g. CT-states). Thus comparable EPC magnitudes for electrons and holes lead to a compensation effect in the Gaussian broadening of the molecular excitons in SubNc, SubPc and Cl_6-SubPc. A further example is found in the EDOS simulation of PTB7-Th:IEICO, where we also predict a large compensation effect as discussed in the main text.

EC reduces linewidth losses in SubNc:Cl_6-SubPc, SubPc:Cl_6-SubPc and neat SubPc – In this paragraph, we discuss how the electronic coupling and consequently the coupling of molecular excitons and CT-excitons affect the linewidth losses and the shape of the low-energy absorption tail.

The material parameters for the EC are calculated with the method presented above. For the SubNc dimers we obtained 8 relevant transfer integrals for the HOMO and the 2-fold degenerate LUMO with maximum size of 35 meV and 40 meV, respectively. The average transfer integral for the LUMOs for all 8

neighbors is $\overline{\varepsilon_{ll'}}$ =22 meV ($\overline{\varepsilon_{hh'}}$ =27 meV for the HOMOs) which is larger than the corresponding C_{60} value, thus, indicating that the EC of neat SubNc is increased compared to the donor:C_{60} blends.

For SubPc we find 10 relevant transfer integrals for the HOMO and the 2-fold degenerate LUMO with 74 meV and 49 meV as the maximum values. The average transfer integrals are $\overline{\varepsilon_{ll'}}$ =22 meV ($\overline{\varepsilon_{hh'}}$ =14 meV for the HOMO) and indicate a slightly smaller EC as compared to SubNc.

Finally, we calculate the EC of neat Cl_6-SubPc. The transfer integral for the 2-fold degenerate LUMO with only 2 relevant couplings to its neighbors is 12 meV (13 meV for the HOMO) which is much smaller than for the SubNc and SubPc leading to a very small EC.

In the EDOS simulations of SubNc:Cl_6-SubPc and SubPc:Cl_6-SubPc planar heterojunctions and of neat SubPc (see upper three panels of Fig. 5b in the main text) we obtained exponential tail shapes in contrast to the mainly Gaussian linewidth of the C_{60}-based blends. For SubNc:Cl_6-SubPc we extract a very small Urbach energy of E_u = 21.6 meV (24.5 meV for SubPc:Cl_6-SubPc and 20.5 meV for neat SubPc) and predict linewidth losses of ΔV_r = 42.5 mV (64.8 mV for Cl_6-SubPc and 37.7 mV for neat SubPc) as compared to the SQ limit. The comparison to the experimental linewidth losses (see Fig. 5a of the main text) shows a very good quantitative agreement for the absorption tails of all three systems which are dominated by the intrinsic absorption of the neat materials SubNc, SubPc and Cl_6-SubPc. We find that the strength of EC manifests in the formation of exponential tails as a signature of the onset of delocalisation of the molecular exciton states in the neat compounds in full consistency with the proposed reduction strategies.

The obtained data for the EC supports our third design strategy that large EC (e.g. by a large number of transfer integrals) may further reduce the EPC induced broadening leading to very small linewidth-induced voltage losses for both molecular- and CT-excitons. This suggests that SubNc, Cl_6-SubPc and SubPc are superior to C_{60} when used in organic solar cells. A further example that confirms our findings is the EDOS simulation of the planar heterojunction of rubrene:C_{60}, where the effect of large EC is directly visible in the CT-states as discussed in the main text.

SM.7 Homogeneous broadening of an excitonic state in the single-state limit

Homogeneous broadening of an excitonic-state and of the zero-phonon line - The broadening of low-energy tails due to low-energy vibrations that we study in organic solar cell films is derived from molecular

vibrations and is additionally affected by the electronic coupling (EC) to the surrounding host. If the surrounding is homogeneous, the broadness of the tails is everywhere the same in the sample, hence the denotation *homogeneous*. A different quantity (although also of *homogeneous* type) is the molecular homogeneous linewidth of the zero-phonon line (ZPL) that can be measured for single molecules close to zero Kelvin[59]. The difference is explained in detail here below.

Analytical result for the homogeneous broadening in the single-state limit - To clarify the difference, we consider the limit of vanishing electronic coupling to the surrounding, i.e., an isolated excitonic state in the low-energy absorption spectrum. We will demonstrate that even for very low temperatures (here for 4 K) we find an effective homogeneous broadening for excitations of that state that is induced by the EPC and which can be of several tens of meV. This is in contrast to the homogeneous broadening of the ZPL that can be below 1 GHz for some molecules.

In the single-state limit, the single-particle spectral function $D'(\omega)$ (density of states), according to Feynman's disentangling theorem[60] for operators, is given by the well-known form[61]

$$D'(\omega) = \frac{n_{l,1}}{2\pi\hbar}\int_{-\infty}^{\infty} \mathrm{d}t \exp\left(\frac{i[\hbar\omega + E_l - \Delta_l]t}{\hbar}\right)\Phi_l(t), \qquad (63)$$

with the electronic onsite energy E_l of the LUMO (for the HOMO in full analogy) and the reorganisation energy Δ_l. The time dependent correlation function $\Phi_l(t)$ simplifies in the present case of dispersionless vibrational modes with circular frequency ω_λ (mode index λ) and intra-molecular coupling constants $(g_{ll}^{\lambda})^2$ to

$$\Phi_l(t) = \exp\left(-\sum_\lambda (g_{ll}^{\lambda})^2 \Gamma_\lambda(t)\right), \qquad (64)$$

with

$$\Gamma_\lambda(t) = [1 - \exp(-i\omega_\lambda t)](1 + \langle N_\lambda\rangle) + [1 - \exp(i\omega_\lambda t)]\langle N_\lambda\rangle. \qquad (65)$$

It depends only on the EPCs g_{ll}^{λ} and the mode energy ω_λ of the set of vibrations λ, which are calculated from DFT (see section SM.3 in the Supplementary Methods for details). The reorganization energy Δ_l is then composed of the reorganisation energy of the individual modes $\Delta_l = \sum_\lambda \Delta_\lambda$. The result of the time integration in Supplementary Equation 63 leads to the analytical expression

$$D'(\omega) = n_{l,1} \sum_{n_1=-\infty}^{\infty} \dots \sum_{n_\lambda=-\infty}^{\infty} \prod_\lambda [W(\lambda, n_\lambda, \beta, \langle N_\lambda\rangle)]\, \delta(\hbar\omega + E_l - \Delta_l - \sum_\lambda \hbar\omega_\lambda n_\lambda), \qquad (66)$$

which is a series of discrete peaks for each superposition of the energies $\sum_\lambda \hbar\omega_\lambda n_\lambda$ weighted with the temperature dependent factors

$$W(\lambda, n_\lambda, \beta, \langle N_\lambda\rangle) = \exp\left(-\left(g_{ll}^{\lambda}\right)^2 (1 + 2\langle N_\lambda\rangle)\right) I_{n_\lambda}\left(2\left(g_{ll}^{\lambda}\right)^2 \sqrt{\langle N_\lambda\rangle(1 + \langle N_\lambda\rangle)}\right) \exp\left(\frac{\beta\hbar\omega_\lambda n_\lambda}{2}\right) \qquad (67)$$

that include the modified Bessel functions $I_{n_\lambda}\left(2\left(g_{ll}^{\lambda}\right)^2\sqrt{\langle N_\lambda\rangle(1+\langle N_\lambda\rangle)}\right)$ that are evaluated at the given temperature with the thermal occupation $\langle N_\lambda\rangle$ of mode λ. The product of these functions leads to the spectral function in the single-state limit. This is an exact result at vanishing electronic coupling.

Analogously, for the isolated excitonic state that we consider as a model, we find the excitonic two-particle spectral function (EDOS)

$$D(\omega) = \frac{n_{lh,1}}{2\pi\hbar}\int_{-\infty}^{\infty} \mathrm{d}t \exp\left(\frac{i[\hbar\omega+E_{\mathrm{CT}}]t}{\hbar}\right)\Phi_{lh}(t) \tag{68}$$

with the energy of the state $E_{\mathrm{CT}} = E_{ll} - \Delta_{ll} + E_{hh} - \Delta_{hh} - V_{hllh}$ and the correlation function

$$\Phi_{lh}(t) = \exp\left(-\sum_\lambda (g_{ll}^{\lambda})^2\Gamma_\lambda(t) - \sum_\lambda (g_{hh}^{\lambda})^2\Gamma_\lambda(t)\right). \tag{69}$$

$\Phi_{lh}(t)$ describes the phase dynamics of a LUMO electron at site l and a HOMO hole at site h. The analytical expression for the spectral function in the single-state limit is the convolution of contributions from all intra-molecular modes of the associated donor (D) and acceptor (A) molecules according to

$$D(\omega) = n_{lh,1}\sum_{n_1=-\infty}^{\infty}\cdots\sum_{n_\lambda=-\infty}^{\infty}\prod_{\lambda \text{ of A,D}}[W(\lambda, n_\lambda, \beta, \langle N_\lambda\rangle)]\,\delta(\hbar\omega + E_{\mathrm{CT}} - \hbar\omega_\lambda n_\lambda), \tag{70}$$

indicated by the mode index λ that, in the case of a CT state, runs over donor and acceptor molecular modes.

In Supplementary Figure 1 we calculate $D(\omega)$ in this limit (isolated excitonic state) based on the obtained EPC constants for the four D-A blends (rubrene:C_{60}, m-MTDATA:C_{60}, Spiro-MeO-TPD:C_{60} and TAPC:C_{60}) whose temperature dependent linewidths have been studied in the main text. The temperature is set to 4 K which is also the case in the single-molecule experiments[59]. The obtained spectra for the isolated excitonic states are either composed of many individual but a dense set of discrete peaks (rubrene:C_{60} and TAPC:C_{60}) or of a continuous distribution of resonances over energy (m-MTDATA:C_{60} and Spiro-MeO-TPD:C_{60}). The observed absorption lines are caused by the combination of multi-phonon absorption processes in the rich manifold of molecular modes in accordance to the analytical expression above. The comparison of the four spectra in Supplementary Figure 1 shows that the lower the energy of the strongly electron-phonon coupled modes the closer are the discrete peaks. We observe that a continuous situation can easily occur for a moderate number of low-frequency modes.

Supplementary Figure 1 illustrates the different linewidths under discussion. The multiple vibrational sub-lines of the considered isolated transition create an effective broadening. In dependence on the density of the electron-phonon coupled low-energy vibrations, it can be an effective broadening of a continuous distribution. We note that the transition between a dense set of discrete peaks (rubrene:C60) and a continuous distribution is triggered by an increasing number of low-frequency modes. In addition, a more continuous distribution is observed with finite temperatures above 80 K or even at room temperature

with a broadness that is the broadness $\sigma_{\text{loc}}(T)$ in Supplementary Equation 18 induced by the thermal disorder potential $V(T)$. In addition, the transition to a continuous distribution is much more likely if (beyond the single state model considered here) additional transfer integrals are considered as in the organic thin films studied in the main text. Because of these transfer integrals in the thin film, one cannot observe any discreteness in the spectra for none of the systems. This effective broadening is the relevant quantity for absorption in organic solar cells since the entire broadened electronic transition absorbs the light. Hence, we call it the broadening due to zero-point vibrations in this work.

From the standpoint of single-molecular spectroscopy, this might be termed differently (e.g. 'effective broadening') because it is to be distinguished from the homogeneous broadening of the zero-phonon line (ZPL) which describes the broadness of an individual peak associated to a zero-phonon (0-0) transition between the vibrational ground states of all modes. The latter can be several orders of magnitude smaller (GHz or even tens of MHz).[59] From the spectra in Supplementary Figure 1 one can understand that only for specific molecules the broadening of the ZPL can be observed, namely for those with a very sparse vibration spectrum of strong coupling modes. Even for the TAPC:C_{60} case it is not visible. In contrast, for the molecules used here the denser vibrational spectrum creates a more continuous absorption spectrum (see above paragraph).

We emphasize that even in the case of the considered isolated transitions and ultralow temperatures it is generally very difficult to observe a ZPL and its linewidth, which was achieved recently[59]. In the context of the present work, this is not relevant because it is many orders of magnitude smaller. Instead, we need to consider the low-frequency-modes-induced effective linewidth seen in Supplementary Figure 1 because we are concerned with the absorption properties of the organic film, which is relevant for the radiative voltage loss.

SM.8 Molecular insights into EPC and tuning thereof

Generally speaking, the electron-phonon coupling is strongly connected to the structure of the considered molecular orbital and its impact on the molecular structure. For instance, when charging a molecule negatively, the deformation of the molecule from its neutral relaxed state to its anionic relaxed state is dominantly affected by the singly occupied LUMO. At bonding lobes of the orbital, bond lengths are shortened, whereas at anti-bonding nodes, bond lengths are increased. These deformations can be

described by combining vibrational normal modes with the EPC values characterizing the energetic contribution to the relaxation of the individual normal modes.

These basic considerations show the connection between the molecular structure and the EPC values and led to design strategies proposed in several systematic theoretical studies. In general, organic molecules can be roughly classified into three groups: (i) molecules with dominant high-frequency modes, (ii) molecules with dominant low-frequency modes, and (iii) molecules with a balanced contribution of normal modes over the entire frequency range[9]. Class (i) includes mostly relatively compact molecules with the orbital being delocalised over a π-conjugated core structure, such as rubrene. This behaviour applies to the entire class of oligoacenes, where the EPC values decrease with increasing number of phenyl rings[29]. In fact, orbital delocalisation is an established design strategy for reduced EPC values over the entire frequency range as the deformation of the molecules upon charging is reduced and the effect of a single molecular orbital on the molecular geometry is weakened[62]. Such a manipulation of the molecular structure also leads to smaller contributions by zero-point vibrations.

However, an extended π-conjugated core structure does not necessarily prevent from contributions by low-frequency modes as demonstrated for more discotic molecules such as triphenylene, perylene, or hexabenzocoronene[29,63,64]. The transition from group (i) to group (ii) is gradual. An increased flexibility of the molecular core leads to increased EPC values. This is also observed when comparing pentacene with thiophene-based analogues[65].

The effect of flexibility is even more pronounced when attaching side chains to the molecular core. When comparing the EPC values of tetracene with the ones of rubrene, one observes noticeable contributions by low-frequency modes by side-group bending vibrations, although the frontier molecular orbitals are widely restricted to the tetracene core[66]. This is even more pronounced for molecules with distinct orbital delocalisation over flexible side groups as demonstrated for the material class of aza-BODIPYs[62,67,68]. Increased orbital delocalisation can decrease the EPC values, however, the side groups especially induce additional contributions by low-frequency vibrations. It has been demonstrated for this material class that the design strategy of rigidification can lead to a significant reduction of EPC values. There are multiple avenues for reducing the flexibility of the side groups including the use of intramolecular hydrogen bonds, steric hindrances, or covalent bridges.

Molecules of group (ii) with dominant EPC values by low-frequency modes often exhibit a torsionally flexible molecular core with delocalised molecular orbitals, such as m-MTDATA or oligothiophenes[69]. Also for these molecules, delocalisation of the orbitals can effectively reduce the EPC values over the entire

frequency range. When comparing m-MTDATA and Spiro-MeO-TPD, also a rigidification of the central part of the molecule can lead to an improvement.

It needs to be highlighted that even the attachment of small side groups, such as methyl or methoxy groups, can increase the EPC values[62,63,70].

Supplementary References

1. Vandewal, K. Interfacial Charge Transfer States in Condensed Phase Systems. *Annu. Rev. Phys. Chem.* **67**, 113–133 (2016).
2. Liu, X., Yi, S., Wang, C., Wang, C. & Gao, Y. Electronic structure evolution and energy level alignment at C_{60} /4,4′-cyclohexylidenebis[N,N-bis(4-methylphenyl) benzenamine]/MoO_x /indium tin oxide interfaces. *J. Appl. Phys.* **115**, 163708 (2014).
3. Polander, L. E. *et al.* Hole-transport material variation in fully vacuum deposited perovskite solar cells. *APL Mater.* **2**, 081503 (2014).
4. Braun, S., de Jong, M. P., Osikowicz, W. & Salaneck, W. R. Influence of the electrode work function on the energy level alignment at organic-organic interfaces. *Appl. Phys. Lett.* **91**, 202108 (2007).
5. Borsenberger, P. M. & Bässler, H. Concerning the role of dipolar disorder on charge transport in molecularly doped polymers. *J. Chem. Phys.* **95**, 5327–5331 (1991).
6. Grover, R., Srivastava, R., Dagar, J., Kamalasanan, M. N. & Mehta, D. S. Interface modified thermally stable hole transporting layer for efficient organic light emitting diodes. *J. Appl. Phys.* **116**, 063102 (2014).
7. Chauhan, G. *et al.* Frequency dependent electrical transport properties of 4,4′,4″-tris(N-3-methylphenyl-N-phenylamine)triphenylamine by impedance spectroscopy. *Synth. Met.* **160**, 1422–1426 (2010).
8. Takahashi, T. *et al.* Gas and Solid Phase Photoelectron Spectra of 5,6,11,12-Tetra-phenylnaphthacene (Rubrene). *Bull. Chem. Soc. Jpn.* **52**, 380–382 (1979).
9. Vandewal, K. *et al.* Absorption Tails of Donor:C_{60} Blends Provide Insight into Thermally Activated Charge-Transfer Processes and Polaron Relaxation. *J. Am. Chem. Soc.* **139**, 1699–1704 (2017).
10. Qian, D. *et al.* Design rules for minimizing voltage losses in high-efficiency organic solar cells. *Nat. Mater.* **17**, 703–709 (2018).
11. Fusella, M. A. *et al.* Band-like Charge Photogeneration at a Crystalline Organic Donor/Acceptor Interface. *Adv. Energy Mater.* **8**, 1701494 (2018).
12. Ordejón, P., Boskovic, D., Panhans, M. & Ortmann, F. *Ab initio* study of electron-phonon coupling in rubrene. *Phys. Rev. B* **96**, 035202 (2017).
13. Haydock, R., Heine, V. & Kelly, M. J. Electronic structure based on the local atomic environment for tight-binding bands. *J. Phys. C Solid State Phys.* **5**, 2845–2858 (1972).

14. Ortmann, F., Cresti, A., Montambaux, G. & Roche, S. Magnetoresistance in disordered graphene: The role of pseudospin and dimensionality effects unraveled. *EPL* **94**, 47006 (2011).

15. Heiney, P. A. *et al.* Orientational ordering transition in solid C60. *Phys. Rev. Lett.* **66**, 2911–2914 (1991).

16. Gaul, C. *et al.* Insight into doping efficiency of organic semiconductors from the analysis of the density of states in n-doped C60 and ZnPc. *Nat. Mater.* **17**, 439–444 (2018).

17. Ortmann, F., Hannewald, K. & Bechstedt, F. *Ab initio* description and visualization of charge transport in durene crystals. *Appl. Phys. Lett.* **93**, 222105 (2008).

18. Bakulin, A. A. *et al.* The role of driving energy and delocalized States for charge separation in organic semiconductors. *Science* **335**, 1340–1344 (2012).

19. Gélinas, S. *et al.* Ultrafast long-range charge separation in organic semiconductor photovoltaic diodes. *Science* **343**, 512–516 (2014).

20. Kwiatkowski, J. J., Frost, J. M. & Nelson, J. The Effect of Morphology on Electron Field-Effect Mobility in Disordered C60 Thin Films. *Nano Lett.* **9**, 1085–1090 (2009).

21. Shirley, E. L. & Louie, S. G. Electron excitations in solid C60: Energy gap, band dispersions, and effects of orientational disorder. *Phys. Rev. Lett.* **71**, 133–136 (1993).

22. Lof, R. W., van Veenendaal, M. A., Koopmans, B., Jonkman, H. T. & Sawatzky, G. A. Band gap, excitons, and Coulomb interaction in solid C60. *Phys. Rev. Lett.* **68**, 3924–3927 (1992).

23. Gaussian 09, Revision A.02, Frisch, M. J. et al., Gaussian, Inc., W. C. Gaussian 09. (2016).

24. Becke, A. D. Density-functional exchange-energy approximation with correct asymptotic behavior. *Phys. Rev. A* **38**, 3098–3100 (1988).

25. Becke, A. D. A new mixing of Hartree–Fock and local density-functional theories. *J. Chem. Phys.* **98**, 1372–1377 (1993).

26. Becke, A. D. Density-functional thermochemistry. III. The role of exact exchange. *J. Chem. Phys.* **98**, 5648–5652 (1993).

27. Krishnan, R., Binkley, J. S., Seeger, R. & Pople, J. A. Self-consistent molecular orbital methods. XX. A basis set for correlated wave functions. *J. Chem. Phys.* **72**, 650–654 (1980).

28. Valiev, M. *et al.* NWChem: A comprehensive and scalable open-source solution for large scale molecular simulations. *Comput. Phys. Commun.* **181**, 1477–1489 (2010).

29. Ortmann, F. *et al.* Materials Meets Concepts in Molecule-Based Electronics. *Adv. Funct. Mater.* **25**, 1933–1954 (2015).

30. Bredas, J.-L. Mind the gap! *Mater. Horiz.* **1**, 17–19 (2014).

31. Wu, Q. & Van Voorhis, T. Direct optimization method to study constrained systems within density-functional theory. *Phys. Rev. A* **72**, 024502 (2005).

32. Binkley, J. S., Pople, J. A. & Hehre, W. J. Self-consistent molecular orbital methods. 21. Small split-valence basis sets for first-row elements. *J. Am. Chem. Soc.* **102**, 939–947 (1980).

33. Yanai, T., Tew, D. P. & Handy, N. C. A new hybrid exchange–correlation functional using the Coulomb-attenuating method (CAM-B3LYP). *Chem. Phys. Lett.* **393**, 51–57 (2004).

34. Grimme, S., Antony, J., Ehrlich, S. & Krieg, H. A consistent and accurate *ab initio* parametrization of density functional dispersion correction (DFT-D) for the 94 elements H-Pu. *J. Chem. Phys.* **132**, 154104 (2010).

35. van Leeuwen, R. & Baerends, E. J. Exchange-correlation potential with correct asymptotic behavior. *Phys. Rev. A* **49**, 2421–2431 (1994).

36. Huang, D.-L., Dau, P. D., Liu, H.-T. & Wang, L.-S. High-resolution photoelectron imaging of cold C60− anions and accurate determination of the electron affinity of C_{60}. *J. Chem. Phys.* **140**, 224315 (2014).

37. Hebard, A. F., Haddon, R. C., Fleming, R. M. & Kortan, A. R. Deposition and characterization of fullerene films. *Appl. Phys. Lett.* **59**, 2109–2111 (1991).

38. Elstner, M. & Seifert, G. Density functional tight binding. *Philos. Trans. R. Soc. A Math. Phys. Eng. Sci.* **372**, 20120483–20120483 (2014).

39. Aradi, B., Hourahine, B. & Frauenheim, T. DFTB+, a Sparse Matrix-Based Implementation of the DFTB Method [†]. *J. Phys. Chem. A* **111**, 5678–5684 (2007).

40. Yang, Yu, H., York, D., Cui, Q. & Elstner, M. Extension of the Self-Consistent-Charge Density-Functional Tight-Binding Method: Third-Order Expansion of the Density Functional Theory Total Energy and Introduction of a Modified Effective Coulomb Interaction. *J. Phys. Chem. A* **111**, 10861–10873 (2007).

41. Gaus, M., Cui, Q. & Elstner, M. DFTB3: Extension of the Self-Consistent-Charge Density-Functional Tight-Binding Method (SCC-DFTB). *J. Chem. Theory Comput.* **7**, 931–948 (2011).

42. Gaus, M., Goez, A. & Elstner, M. Parametrization and Benchmark of DFTB3 for Organic Molecules. *J. Chem. Theory Comput.* **9**, 338–354 (2013).

43. Gaus, M., Cui, Q. & Elstner, M. Density functional tight binding: application to organic and biological molecules. *Wiley Interdiscip. Rev. Comput. Mol. Sci.* **4**, 49–61 (2014).

44. Zhechkov, L., Heine, T., Patchkovskii, S., Seifert, G. & Duarte, H. A. An Efficient *a Posteriori* Treatment for Dispersion Interaction in Density-Functional-Based Tight Binding. *J. Chem. Theory*

Comput. **1**, 841–847 (2005).

45. Nosé, S. A unified formulation of the constant temperature molecular dynamics methods. *J. Chem. Phys.* **81**, 511–519 (1984).
46. Hoover, W. G. Canonical dynamics: Equilibrium phase-space distributions. *Phys. Rev. A* **31**, 1695–1697 (1985).
47. Martyna, G. J., Tuckerman, M. E., Tobias, D. J. & Klein, M. L. Explicit reversible integrators for extended systems dynamics. *Mol. Phys.* **87**, 1117–1157 (1996).
48. Vandewal, K., Tvingstedt, K., Gadisa, A., Inganäs, O. & Manca, J. V. On the origin of the open-circuit voltage of polymer–fullerene solar cells. *Nat. Mater.* **8**, 904–909 (2009).
49. Vandewal, K., Tvingstedt, K., Gadisa, A., Inganäs, O. & Manca, J. V. Relating the open-circuit voltage to interface molecular properties of donor:acceptor bulk heterojunction solar cells. *Phys. Rev. B* **81**, 125204 (2010).
50. Rau, U. Reciprocity relation between photovoltaic quantum efficiency and electroluminescent emission of solar cells. *Phys. Rev. B* **76**, 085303 (2007).
51. Benduhn, J. *et al.* Intrinsic non-radiative voltage losses in fullerene-based organic solar cells. *Nat. Energy* **2**, 17053 (2017).
52. Endres, J., Pelczer, I., Rand, B. P. & Kahn, A. Determination of Energy Level Alignment within an Energy Cascade Organic Solar Cell. *Chem. Mater.* **28**, 794–801 (2016).
53. Sullivan, P. *et al.* Halogenated Boron Subphthalocyanines as Light Harvesting Electron Acceptors in Organic Photovoltaics. *Adv. Energy Mater.* **1**, 352–355 (2011).
54. Dang, J. D. *et al.* The mixed alloyed chemical composition of chloro-(chloro)$_n$-boron subnaphthalocyanines dictates their physical properties and performance in organic photovoltaic devices. *J. Mater. Chem. A* **4**, 9566–9577 (2016).
55. Fulford, M. V. *et al.* Crystal Structures, Reaction Rates, and Selected Physical Properties of Halo-Boronsubphthalocyanines (Halo = Fluoride, Chloride, and Bromide). *J. Chem. Eng. Data* **57**, 2756–2765 (2012).
56. Morse, G. E., Gong, I., Kawar, Y., Lough, A. J. & Bender, T. P. Crystal and Solid-State Arrangement Trends of Halogenated Boron Subphthalocyanines. *Cryst. Growth Des.* **14**, 2138–2147 (2014).
57. Baumeier, B., Kirkpatrick, J. & Andrienko, D. Density-functional based determination of intermolecular charge transfer properties for large-scale morphologies. *Phys. Chem. Chem. Phys.* **12**, 11103 (2010).
58. Gaussian 16, Revision B.01, Frisch, M. J. et al. , Gaussian, Inc., W. C. Gaussian 16. (2016).

59. Wang, D. *et al.* Turning a molecule into a coherent two-level quantum system. *Nat. Phys.* **15**, 483–489 (2019).

60. Feynman, R. P. Mathematical Formulation of the Quantum Theory of Electromagnetic Interaction. *Phys. Rev.* **80**, 440–457 (1950).

61. Duke, C. B. & Mahan, G. D. Phonon-Broadened Impurity Spectra. I. Density of States. *Phys. Rev.* **139**, A1965–A1982 (1965).

62. Schellhammer, K. S. *et al.* Tuning Near-Infrared Absorbing Donor Materials: A Study of Electronic, Optical, and Charge-Transport Properties of aza-BODIPYs. *Chem. Mater.* **29**, 5525–5536 (2017).

63. Gisslén, L. & Scholz, R. Crystallochromy of perylene pigments: Interference between Frenkel excitons and charge-transfer states. *Phys. Rev. B* **80**, 115309 (2009).

64. Vincent Lemaur, † *et al.* Charge Transport Properties in Discotic Liquid Crystals: A Quantum-Chemical Insight into Structure−Property Relationships. (2004).

65. Veaceslav Coropceanu, † *et al.* Charge Transport in Organic Semiconductors. (2007).

66. da Silva Filho, D. A., Kim, E.-G. & Brédas, J.-L. Transport Properties in the Rubrene Crystal: Electronic Coupling and Vibrational Reorganization Energy. *Adv. Mater.* **17**, 1072–1076 (2005).

67. Lorenz-Rothe, M. *et al.* From Fluorine to Fluorene-A Route to Thermally Stable *aza* -BODIPYs for Organic Solar Cell Application. *Adv. Electron. Mater.* **2**, 1600152 (2016).

68. Radke, K. S., Scholz, R., Ortmann, F., Leo, K. & Cuniberti, G. Dynamic Effects on the Charge Transport in an Organic Near-Infrared Absorber Material. *J. Phys. Chem. C* **118**, 6537–6547 (2014).

69. da Silva Filho, D. A. *et al.* Hole-vibronic coupling in oligothiophenes: impact of backbone torsional flexibility on relaxation energies. *Philos. Trans. R. Soc. A Math. Phys. Eng. Sci.* **365**, 1435–1452 (2007).

70. Kraner, S. *et al.* Influence of side groups on the performance of infrared absorbing aza-BODIPY organic solar cells. *Phys. status solidi* **212**, 2747–2753 (2015).
